# Insights on Cancer Cell Inhibition, Subcellular Activities, and Kinase Profile of Phenylacetamides Pending 1*H*-Imidazol-5-One Variants

**DOI:** 10.3389/fphar.2021.794325

**Published:** 2022-01-05

**Authors:** Maan T. Khayat, Abdelsattar M. Omar, Farid Ahmed, Mohammad I. Khan, Sara M. Ibrahim, Yosra A. Muhammad, Azizah M. Malebari, Thikryat Neamatallah, Moustafa E. El-Araby

**Affiliations:** ^1^ Faculty of Pharmacy, Department of Pharmaceutical Chemistry, King Abdulaziz University, Jeddah, Saudi Arabia; ^2^ Faculty of Pharmacy, Department of Pharmaceutical Chemistry, Al-Azhar University, Nasr City, Egypt; ^3^ Center for Artificial Intelligence in Precision Medicines, King Abdulaziz University, Jeddah, Saudi Arabia; ^4^ Center of Excellence in Genomic Medicine Research, King Abdulaziz University, Jeddah, Saudi Arabia; ^5^ Faculty of Applied Medical Sciences, King Abdulaziz University, Jeddah, Saudi Arabia; ^6^ Faculty of Science, Department of Biochemistry, King Abdulaziz University, Jeddah, Saudi Arabia; ^7^ Faculty of Pharmacy, Department of Pharmacology and Toxicology, King Abdulaziz University, Jeddah, Saudi Arabia

**Keywords:** tirbanibulin, src kinase, phosphokinase profiling, structure–properties relationship, imidazolone, scaffold hopping, multi-kinase downregulation, leukemia

## Abstract

Structural changes of small-molecule drugs may bring interesting biological properties, especially in the field of kinase inhibitors. We sought to study tirbanibulin, a first-in-class dual Src kinase (non-ATP competitive)/tubulin inhibitor because there was not enough reporting about its structure–activity relationships (SARs). In particular, the present research is based on the replacement of the outer ring of the biphenyl system of 2-[(1,1′-biphenyl)-4-yl]-*N*-benzylacetamide, the identified pharmacophore of KX chemotype, with a heterocyclic ring. The newly synthesized compounds showed a range of activities in cell-based anticancer assays, agreeing with a clear SAR profile. The most potent compound, (*Z*)-*N*-benzyl-4-[4-(4-methoxybenzylidene)-2-methyl-5-oxo-4,5-dihydro-1*H*-imidazol-1-yl]phenylacetamide (KIM-161), demonstrated cytotoxic IC_50_ values at 294 and 362 nM against HCT116 colon cancer and HL60 leukemia cell lines, respectively. Profiling of this compound (aqueous solubility, liver microsomal stability, cytochrome P450 inhibition, reactivity with reduced glutathione, and plasma protein binding) confirmed its adequate drug-like properties. Mechanistic studies revealed that this compound does not depend on tubulin or Src kinase inhibition as a factor in forcing HL60 to exit its cell cycle and undergo apoptosis. Instead, KIM-161 downregulated several other kinases such as members of BRK, FLT, and JAK families. It also strongly suppresses signals of ERK1/2, GSK-3α/β, HSP27, and STAT2, while it downregulated AMPKα1 phosphorylation within the HL60 cells. Collectively, these results suggest that phenylacetamide-1*H*-imidazol-5-one (KIM-161) could be a promising lead compound for further clinical anticancer drug development.

## Introduction

**FIGURE 1:**
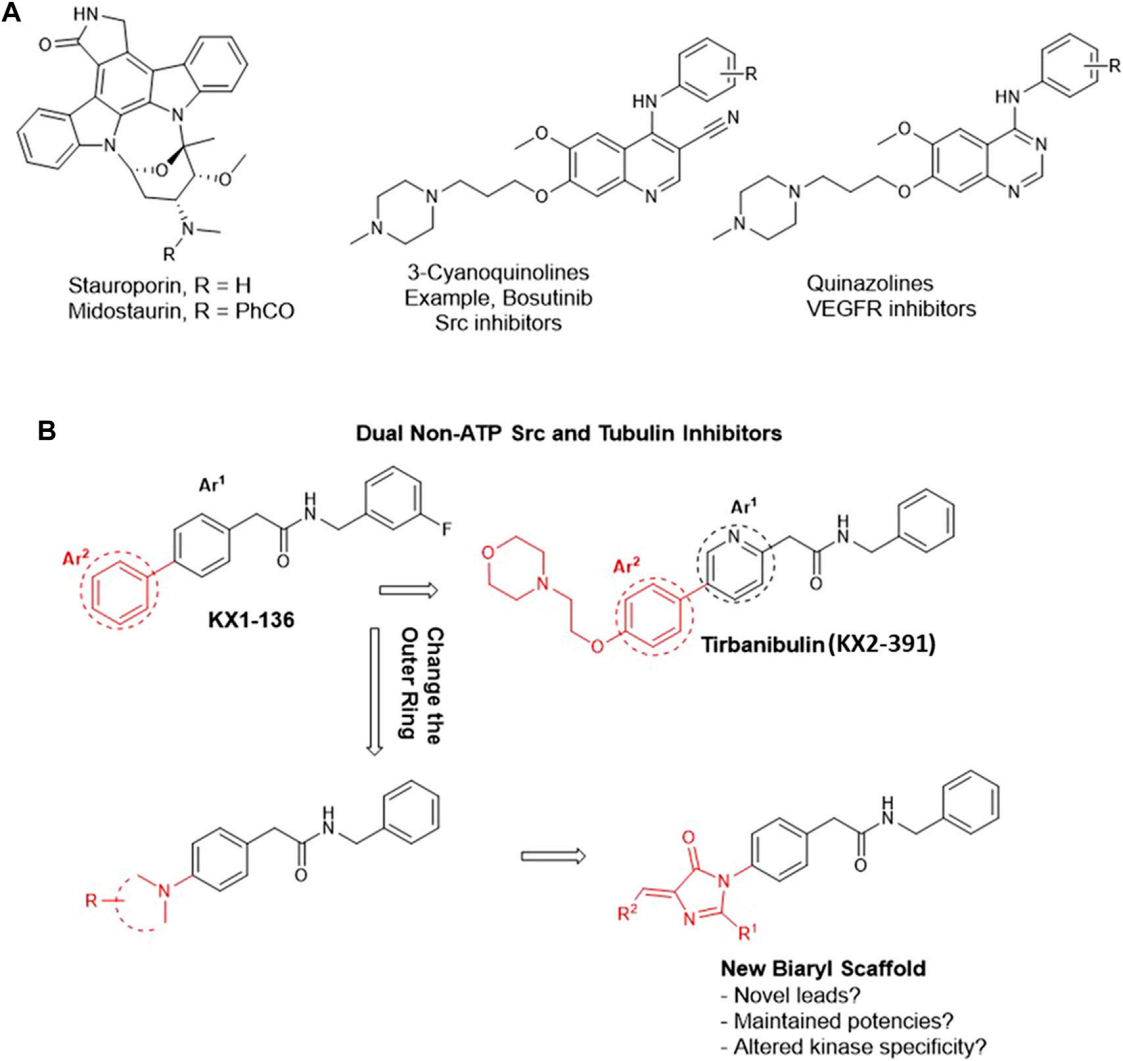
Rational design of imidazolone analogs of KX chemotype. **(A)** Examples of ATP-competitive kinase inhibitors with altered kinase specificity after minor structural changes. Replacing 3-cyanoquinoline with quinazoline caused a shift in kinase inhibition specificity from Src to VEGFR. The benzoyl moiety in midostaurin conferred some specificity toward mutated FLT3 kinase compared to the non-specific staurosporine. **(B)** The black motif is considered a fixed part, common among the prototypes (KX1-136 and KX2-391) and the newly designed compounds. The red parts are variables.

Tirbanibulin (aka KX2-391 and KX-01, Figure 1) is a powerful inhibitor of malignant growth and an approved medication for the treatment of actinic keratosis in the form of an ointment (Klisyri®) (Markham and Duggan, 2021). *In vitro*, KX2-391 demonstrates high cytotoxic activities against a variety of solid and liquid tumor cell lines (Anbalagan et al., 2012, 2017; S. Kim et al., 2017) through dual inhibition of Src kinase (IC_50_ = 25 nM) (Antonarakis et al., 2013) and tubulin polymerization (IC_50_ = 250 nM) (Hangauer, 2007; Smolinski et al., 2018; Markham and Duggan, 2021). The latter mechanism is believed to confer a broad range of cancer cell growth inhibition activities on KX2-391, regardless of its Src dependency/independency (Smolinski et al., 2018). This conclusion is not wrong but, nonetheless, has left some gaps in our understanding of the mechanism of action (MoA) of the KX type of compounds. The high potency of KX2-391 in less Src–expressing cancer cell lines created a perception that the tubulin polymerization inhibition is the sole mechanism involved in the cytotoxic activities in this case. However, this notion remained largely hypothetical as it has never been investigated systemically, and the question of how selective KX2-391 (non-ATP competitive) is toward Src stays unanswered. In a different perspective, the medicinal chemistry of the KX chemotype (2-biaryl-*N*-benzylacetamide) is, until now, limited to a few studies that are not sufficiently informative (Hangauer, 2007; Smolinski et al., 2018). This is relevant to our focus as we think that the structure–activity relationship (SAR) of this drug should be investigated in a broader range to contest the anticancer pharmacophoric constraints in its structure. Another objective of our study was to unravel opportunities to discover interesting anticancer new leads that possibly shifts specificity from Src to other abhorrent oncogenic kinases, just similar to what is commonly observed in ATP-competitive inhibitors (Avendaño and Menéndez, 2015). For instance, changing the bicyclic core of bosutinib (3-cyanoquinoline) to quinazoline shifted the kinase specificity from Src to the vascular endothelial growth factor receptor (VEGFR) (Figure 1A) (Boschelli et al., 2001; Hennequin et al., 2002). Another prominent example is staurosporine, which inhibits most of the tyrosine kinases non-selectively and is therefore clinically irrelevant. Its *N*-benzoyl derivative, midostaurin, tends to have a certain specificity toward mutated FLT3 kinase and has therefore been approved for the treatment of acute myeloid leukemia (AML) leukemia (Figure 1A) (Manley et al., 2018). Nevertheless, it is rare to find a clinically relevant kinase inhibitor that does not bind to other kinases, an approach commonly known as the polypharmacology (multi-targeted) kinase inhibitors (Hartmann et al., 2009). KX2-391 itself was recently found to inhibit a mutated FLT3 kinase (Wang et al., 2021). Therefore, we decided to investigate a change of the biphenyl core of KX series to a related system aiming to: a) study the SAR of KX scaffold extending beyond the biphenyl-4-(*N*-benzyl)acetamide scaffold (KX1-136, Figure 1B), b) monitor the effect of replacing the outer ring of the biaryl system of KX series on kinase activities and specificities, and c) attempt to identify novel molecular leads with potent anticancer activities that are not necessarily dependent on Src/tubulin inhibition (Figure 1B).

In this direction, it should be noted that the few reports discussing SARs of KX2-391 have informed that the presence of a substituted methylene before and after the amide group is crucial for both cellular and Src inhibition activities (Smolinski et al., 2018). In this context, it was observed that a secondary amide group must be preserved in the pharmacophore. The *N*-benzyl ring tolerates no or very limited substitution, such as *m*-fluoro, which in fact is a known hydrogen isostere. The internal Ar^1^ (Figure 1B) should conveniently be either benzene or pyridine. Replacing either of these rings by the isosteric rings such as thiazole, negatively affected the activity (Fallah-Tafti et al., 2011).

Consequently, we fixed the core Ph-CH_2_-CO-NH-CH_2_-Ph and opted to investigate the outer ring Ar^2^ (Figure 1B). The available information about this ring indicated that it tolerated substitutions on the *para* position but no data has been reported about its replacement with radically different moieties (Hangauer, 2007; Smolinski et al., 2018). As an Ar^2^ replacement, we serendipitously selected 5-arylidene-3,5-dihydro-4*H*-imidazol-4-one moiety because it served us successfully to design novel anti-proliferative (Omar et al., 2018), chemosensitizing (El-Araby et al., 2017), and anti-inflammatory (El-Araby et al., 2012) agents. We employed this atypical heteroaromatic motif to bring distinctive receptor interactions compared to the plain phenyl of KX series (Figure 1B), aiming to create possibilities of inhibiting other kinase signals, other than the inhibition of Src kinase.

## Results

### Chemical Synthesis

**SCHEME 1:**
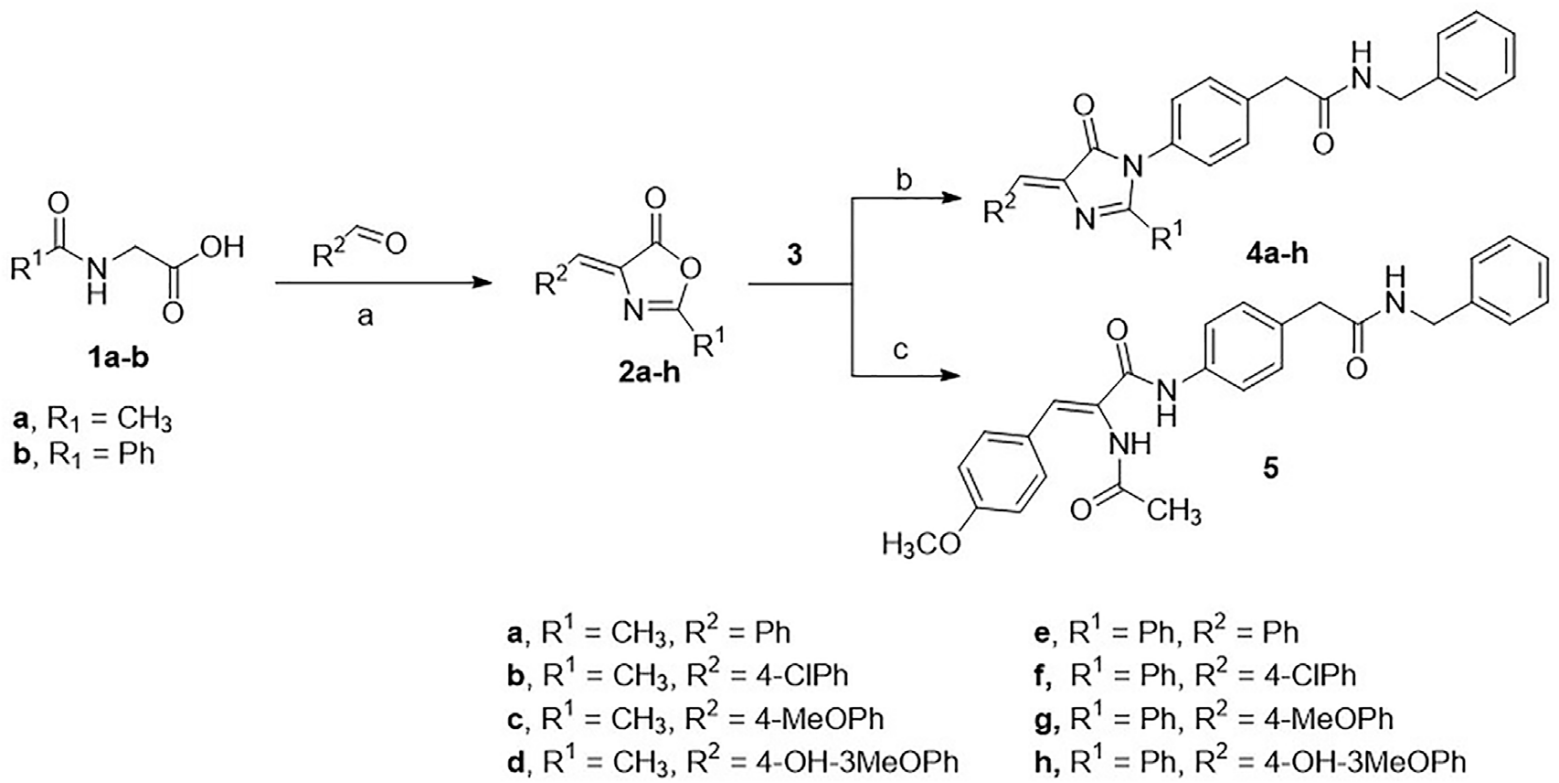
Conditions: **(A)** acetic anhydride, sodium acetate, heat; **(B)** pyridine, heat; **(C)** acetonitrile, heat.

The Erlenmeyer method was adopted to prepare the oxazolone intermediates (**2a-h**) starting from either *N*-acetylglycine or hippuric acid (**1a-b**) and have it react with the appropriate aldehyde. The oxazolones **2a-h** were subjected to cyclo-condensation with 4-amino-*N*-benzylphenyl acetamide **3**) (prepared according to the previously described procedure) (Clark and Davenport, 1987; Maruyama et al., 2009; El-Araby et al., 2020a; El-Araby et al., 2020b) in dry pyridine to produce target compounds **4a-h**. Alternatively, the oxazolone **2c** was heated with **3** in acetonitrile to give the open analog **5** (Scheme 1).

All the compounds were confirmed by spectral analysis using nuclear magnetic resonance (NMR), and the molecular formulae were established using high-resolution mass spectrometry (HRMS). The purities were confirmed using liquid chromatography/mass spectrometry (LC/MS) to be higher than 95%.

### Biological Screening

#### Cytotoxic Effect Against Various Cancer Cell Lines

The compounds were screened for their anticancer activities against a variety of cancer cell lines. The cytotoxic effect of the compounds was evaluated using the sulforhodamine B (SRB) cytotoxicity assay for breast cancer line (MCF7) and prostate cancer cell line (PC3) (Vichai and Kirtikara, 2006; Rana et al., 2021) and 3-(4,5-dimethylthiazol-2-yl)-2,5-diphenyl-2*H*-tetrazolium bromide (MTT) assay for the colon cancer (HCT116) cell lines (El-Huneidi et al., 2020). The CellTiter-Blue® cell viability assay was performed on the various leukemia cell lines, HL60, NB4, and KG1a cells (Akter et al., 2019). The IC_50_ values are illustrated in Table 1.

**TABLE 1:** List of IC_50_ ± SEM values of different compounds against a panel of cancer cell lines.

Compound no.	Project code	IC_50_ ± SEM (µM) MCF7	IC_50_ ± SEM (µM) PC3	IC_50_ ± SEM (µM) HCT116	IC_50_ ± SEM (µM) HL60
**4a**	KIM-111	11.58 ± 2.01	1.667 ± 0.66		
**4b**	KIM-121	20.82 ± 1.35	6.866 ± 0.41	>50	
**4c**	**KIM-161**	1.141 ± 0.19	2.135 ± 0.34	0.294 ± 0.02	0.362 ± 0.02
**4d**	KIM-131	9.481 ± 1.16	5.831 ± 1.44	6.135 ± 1.35	1.435 ± 0.07
**4e**	KIM-211	34.85 ± 8.08	41.68 ± 2.99		
**4f**	KIM-221	44.25 ± 8.67	74.16 ± 12.33		
**4g**	KIM-261	32.07 ± 3.98	38.68 ± 5.01		
**4h**	KIM-231	35.10 ± 4.57	37.51 ± 15.39		
**5**	KIM-161V	44.28 ± 9.20	41.10 ± 9.55		

**FIGURE 2:**
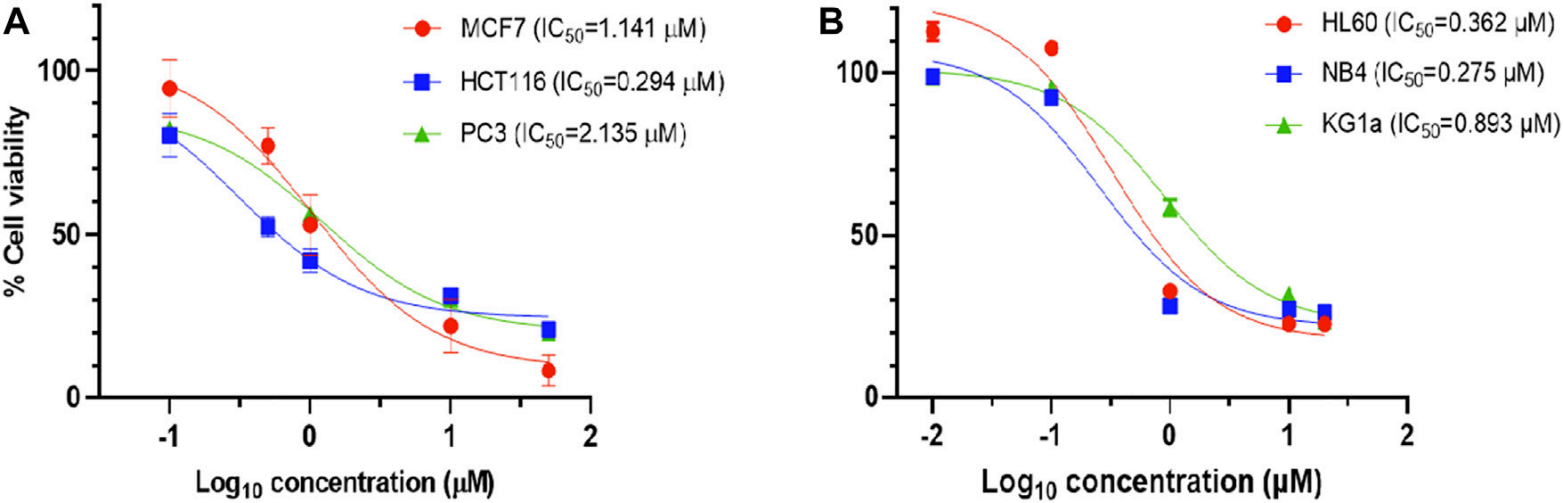
Dose–response curve of 4c (KIM-161) against **(A)** solid tumor cell lines and **(B)** leukemia cell lines.

The results showed that compound **4c** (coded **KIM-161**) attained the highest antiproliferative activities at IC_50_ values ranging from the submicromolar (HCT116 = 0.294 µM, HL60 = 0.362 µM) to the low micromolar level (MCF7 = 1.14 µM, PC3 = 2.13 µM). We extended our cytotoxic screening of KIM-161 against the HCT116 colon cancer cell line and against the NB4 and KG1a leukemia cell lines, and the compound continued to show high activities with IC_50_ values at 0.294, 0.275, and 0.893 µM, respectively (Figure 2). Based on these results, we decided to continue with the HL60 cells for all the downstream experiments. We did not include KX2-391 as a positive reference in this assay and some others because this drug has been extensively studied for its possible mechanism of action(s), while our objective was not to further study it. Rather, we explained above that we are exploring imidazolone as a scaffold hop that may bear a different kinase specificity from KX2-391.

#### Changes in Cell Cycle Distribution After Treatment With KIM-161

**FIGURE 3:**
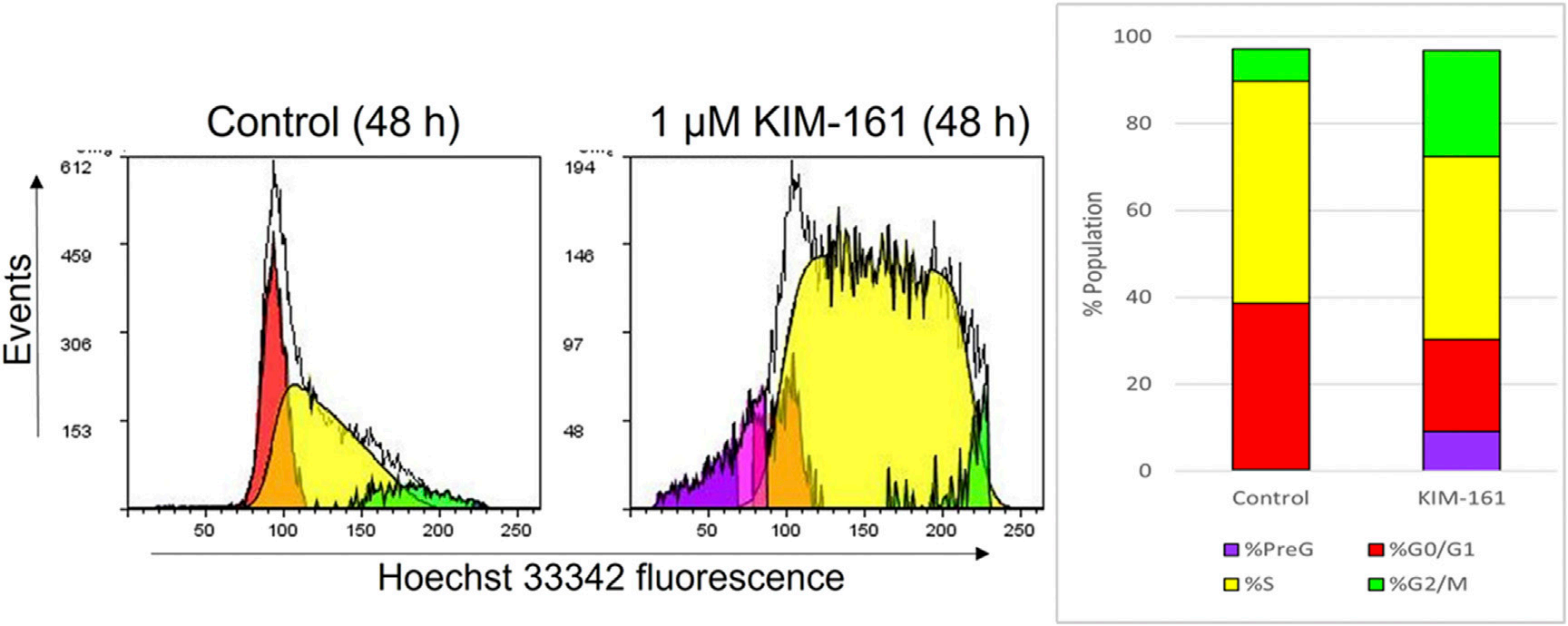
Effect of KIM-161 (1 µM) on HL60 cell cycle compared to untreated cells (control) for 48 h.

The cell cycle analysis of the HL60 cells after treatment was performed with 1 µM of KIM-161 by using flow cytometry. The compound caused the cells to accumulate in PreG phase and increased the population of cells in the G2/M phase. A decrease in the G0/G1 phase is observed after treatment, and no difference is seen in cell population in the S phase when compared to the control (Figure 3).

#### Caspase-3 Activity in Cells After Treatment With KIM-161

**FIGURE 4:**
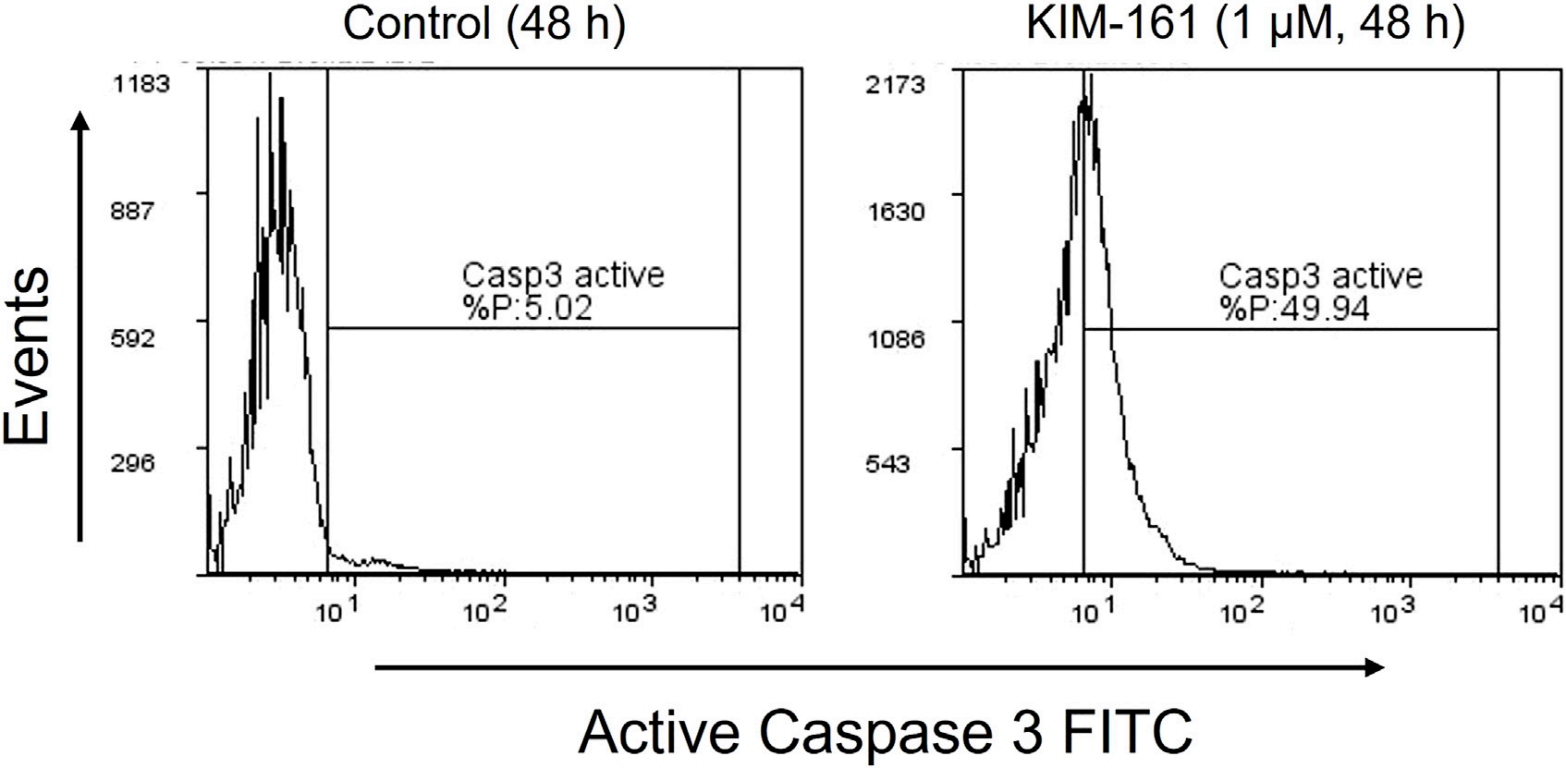
Caspase-3 activity profile after treatment of HL60 cells with KIM-161 (1 µM) compared to untreated cells (control) for 48 h.

The activated caspase-3 is a marker for cell apoptosis that proteolytically cleaves and activates other caspases and intracellular targets (Patel et al., 1996). Caspase-3, which is activated during the early stages of apoptosis, could be measured in HL60 cells undergoing apoptosis by using fluorescein isothiocyanate (FITC)–conjugated active caspase-3 antibody. The treatment of HL60 cells with 1 µM of KIM-161 for 48 h demonstrated approximately a 10-fold increase in the cells undergoing apoptosis as compared to the basal apoptosis of the untreated HL60 cells (Figure 4).

#### Phosphokinase Activity Profiling of KIM-161 in Leukemia Cell Line

The reproducible high potency of KIM-161 on leukemia cell lines was initially speculated to encompass Src tyrosine kinase inhibition. Even so, we also suspected that KIM-161 might be affecting other tyrosine kinases because the HL60 cells are not among the most Src-dependent highly proliferative cells (Collins, 1987). Therefore, a profiling of the kinase activities was performed after treatment with KIM-161 using PamChip® tyrosine kinase microarray. This analysis is based on incubation of cell lysate with 3D microarray that projects peptides specific for 143 tyrosine kinases. If a kinase is modified in the cells, it will be detected by phosphorylating its specific peptide in the microarray.

**FIGURE 5:**
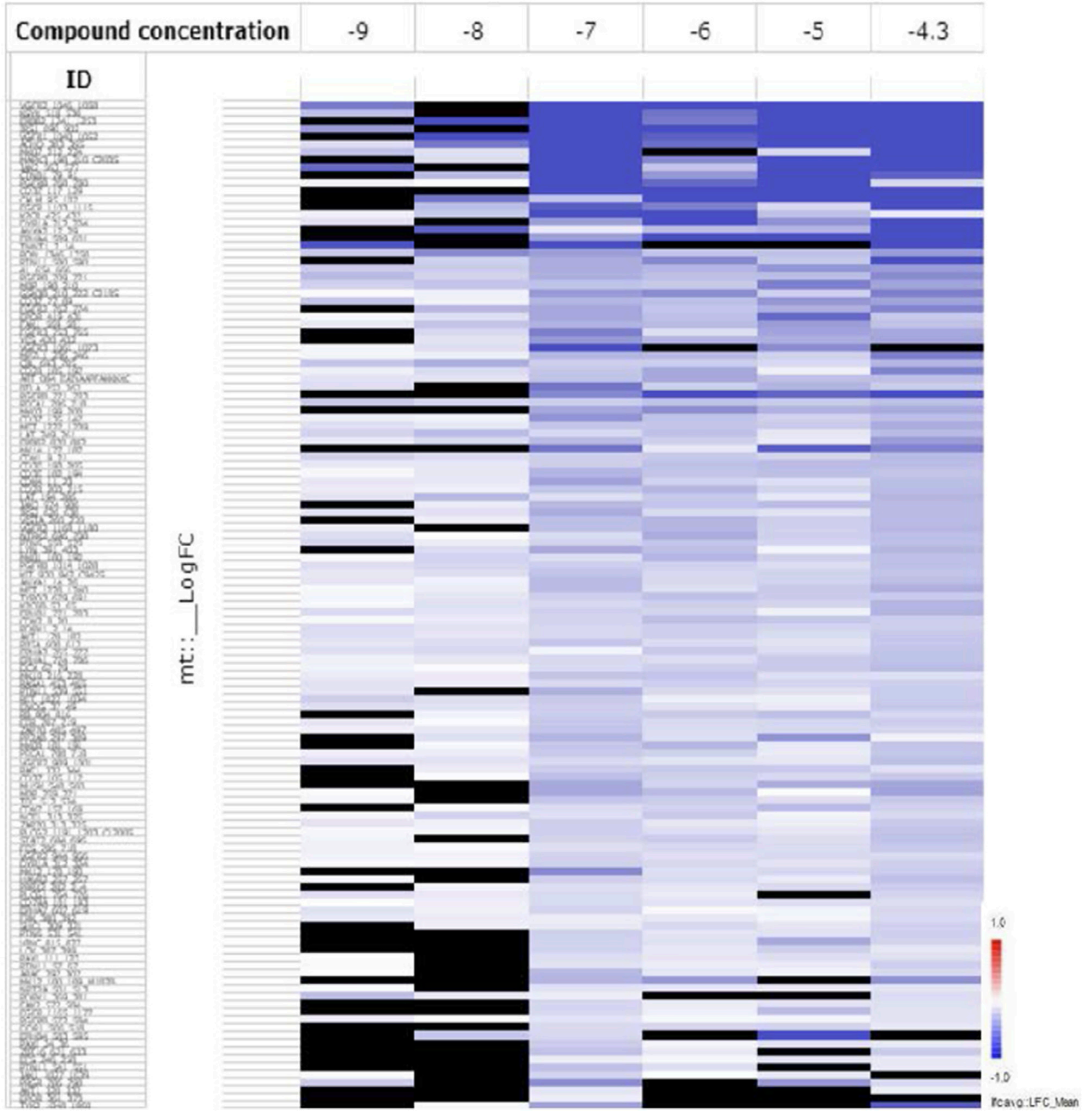
The LFC heat map for peptides shows the values of differentially inhibited phosphorylated peptides (KIM-161 vs. DMSO control). The treatment effects are log-ratios [log2(treatment) − log2(control)]. Black are peptides which did not pass the threshold of inhibition. DMSO concentration 2% v/v; compound concentration log −9 = 0.001 μM; log −8 = 0.01 μM; log −7 = 0.1 μM; log −6 = 1 μM; log −5 = 10 μM, and log −4.3 = 50 μM).

This test measured the activities of an array of tyrosine kinases of treated HL60 cells compared to the control (untreated cells) after incubation with KIM-161 for 48 h at concentrations of 0.001, 0.01, 0.1, 1.0, 10, and 50 µM. The log fold change (LFC) heat map was initially used to identify significant peptides affected at each concentration (Figure 5). The LFC heat map suggested that 0.1 µM was the proper concentration to consider in the analysis of the kinase activity profile of KIM-161.

An Upstream Kinase Tool was used to generate a putative list of kinases responsible for phosphorylating the phosphosites on the PamChip®. It is an interpretation and is highly dependent on the contents of the underlying phosphorylation databases. Kinase score (Table 2) was used for ranking kinases based on their significance and specificity in terms of the set of peptides used for the corresponding kinase. Kinase statistics indicates the overall change of the peptide set that represents a certain kinase. Kinase statistics value of <0 indicates lower kinase activity in the lysate spiked with the compound and vice versa. Table 2 lists the 20 most changed kinases within treated HL60 cell lysates by 0.1 μM KIM-161.

**TABLE 2:** Top 20 affected kinases ranked according to kinase scores for KIM-161 at 0.1 µM treatment of HL60 leukemia cells.

Rank	Kinase name	Kinase score	Kinase statistic
1	SRM	4.10	−1.41
2	ITK	3.95	−1.46
3	JAK1~b	3.51	−1.93
4	FRK	3.45	−1.35
5	Tyro3/Sky	3.29	−1.32
6	FLT1	3.23	−1.41
7	FLT3	3.19	−1.48
8	JAK2	3.19	−1.43
9	FLT4	3.15	−1.45
10	Ret	3.14	−1.45
11	Fes	3.13	−1.47
12	EphA2	3.09	−1.50
13	CSK	3.09	−1.31
14	Ron	2.96	−1.50
15	Ros	2.90	−1.72
16	BTK	2.76	−1.29
17	Lck	2.76	−1.28
18	FGFR4	2.73	−1.26
19	TXK	2.68	−1.25
20	CCK4/PTK7	2.60	−1.44

**FIGURE 6:**
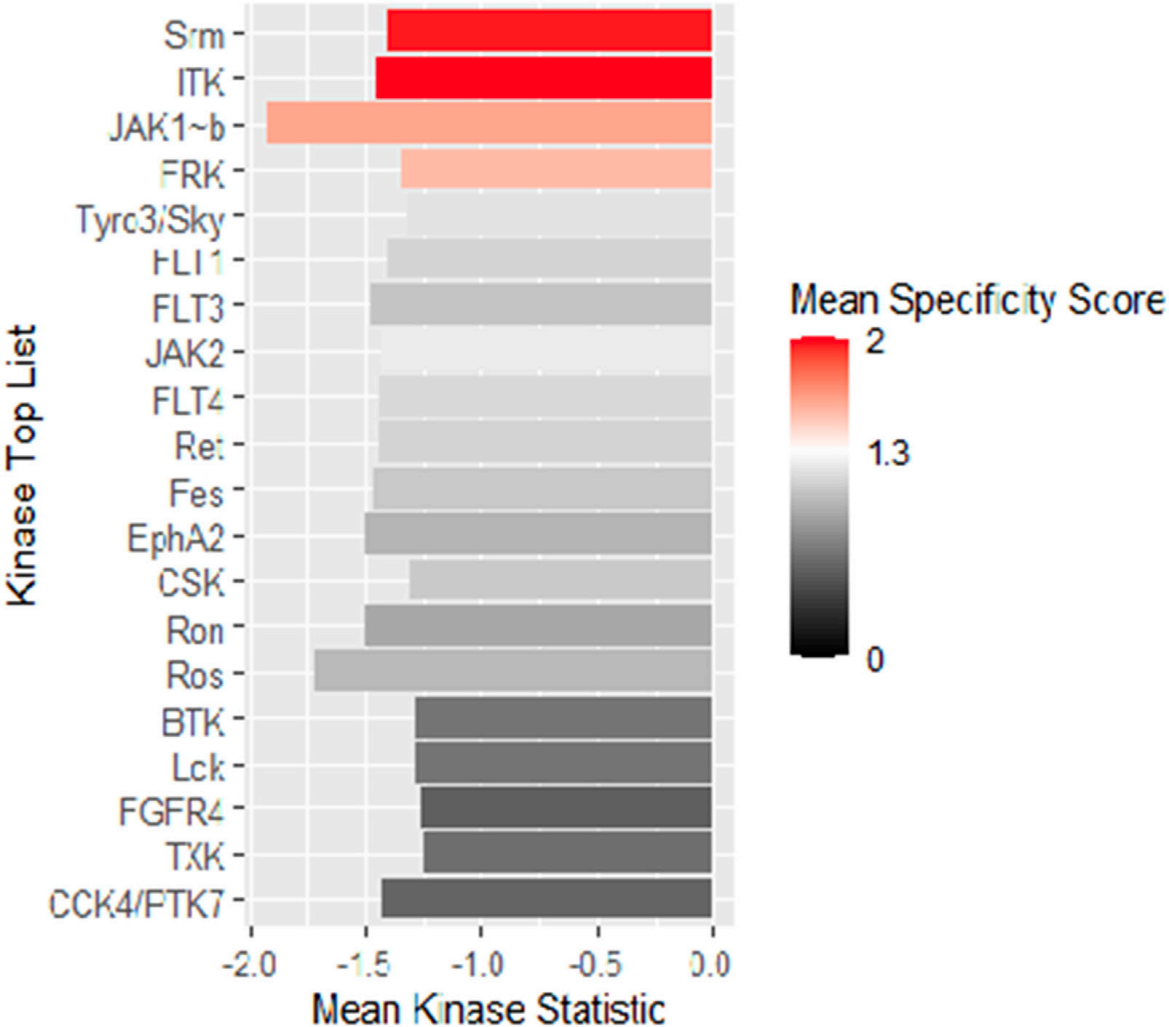
Mean kinase statistics (represented by bar length) and mean specificity score (represented by color scheme) for the top 20 affected PTKs after treatment of HL60 cells with KIM-161 at 0.1 µM.

From the scores and specificities illustrated in Table 2 and Figure 6, it is clear that kinases belonging to the BRK family are among the most affected signaling proteins. For instance, Src-related kinase lacking C-terminal regulatory tyrosine and N-terminal myristylation sites (SRM), a BRK family member (Goel et al., 2018) appeared on the top of this list after combining kinase statistics (−1.14) and specificity scores to provide a kinase score of 4.1. FRK (another BRK family member) occupied the fourth place in the list with a kinase score of 3.45 (Goel and Lukong, 2016). Interestingly, both SRM and FRK belong to a similar subfamily of BRK called SRMs (Goel et al., 2013). Interleukin tyrosine kinase (ITK), a TEC family member (Andreotti et al., 2010), occupied the second most ranked kinase place with altered activities followed by Janus-activated kinase 1 (JAK1) (Reiterer and Yen, 2006). Surprisingly, Src kinase and Src family members barely appeared in the list, as only the lymphocyte-specific protein tyrosine kinase (LCK) was ranked 17th with a kinase score 2.76.

**FIGURE 7:**
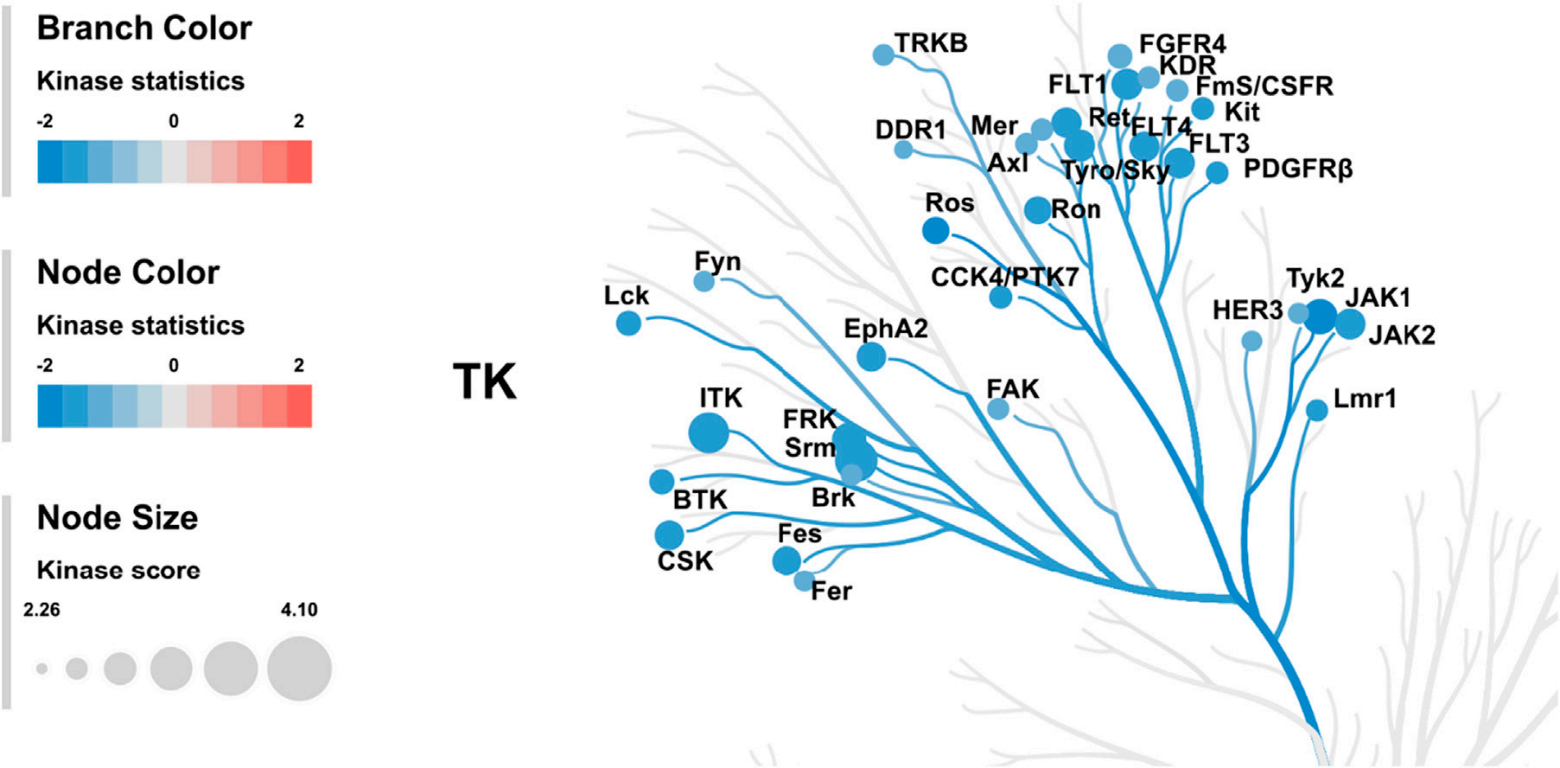
Kinome tree: The top predicted kinases that have been changed after treatment of HL60 cells with KIM-161 are represented on the phylogenetic tree of the human protein kinase family. The size of the dot indicates total kinase score, and the color denotes kinase statistics (red: higher in on-chip–treated lysates, blue: lower in on-chip–treated lysate). Kinase statistics cut-off ≤ −1.2 was applied to select top altered.

In order to generate a ranked list of putative kinases responsible for differences in the peptide phosphorylation, the upstream kinase analysis is used to generate a kinome tree that is useful to group kinases in sequence families (Figure 7) (Metz et al., 2018).

The kinome tree illustrated that KIM-161 forced downregulation of several kinase families such as the BRK family kinases (SRM and FRK), FLT family kinase (VEGFRs), and JAK family kinase. The Src family kinases appeared to be marginally affected (FYN, LCK). Src kinase did not appear in most downregulated tyrosine kinases. In the analysis, it had a kinase score of 2.16 and kinase statistic of 1.07 occupying the 55th place in the 143-tyrosine kinase list mounted on the PamChip®.

**FIGURE 8:**
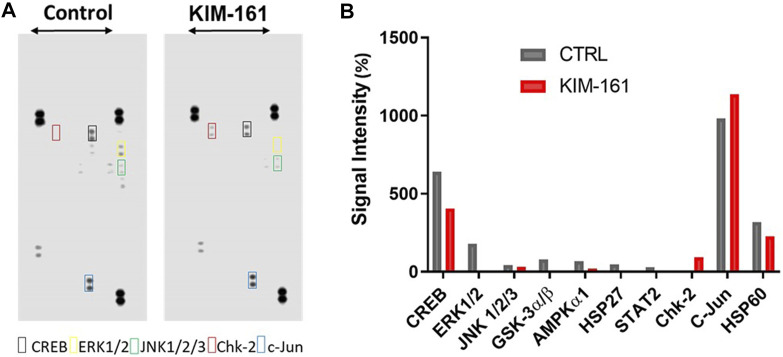
Phosphokinase proteome profile of KIM-161 in HL60 cell line. **(A)** Cells lysates were prepared after 10 h treatment with KIM-161 and applied to the membranes prespotted with the different antibodies. **(B)** The bar graph shows variation of phosphokinases in HL60 cells upon KIM-161 exposure relative to the untreated control cells. The height of each bar represents the average intensity of certain protein signals in treated cells/untreated × 100. Therefore, the dotted line represents the control percentage (100%).

We also measured changes in kinase activities of some selected Ser/Thr kinases using the Human Phospho-Kinase Array® kit (R&D Systems) which encompasses phosphoprotein-specific capture antibodies spotted at a certain location (in duplicates) on a nitrocellulose membrane. The signal intensities, detected by a sandwich immunoassay, are proportional to the amount of each phosphokinase in the cell lysate (Figure 8).

In the analysis, we took into consideration the relative intensity of different kinases in the untreated HL60 cells (control) because this can be taken as an indicator of the importance of this specific phosphoprotein in the survival and proliferation of the cancer. Therefore, the compound was found to cause strong downregulation of ERK1/2 (T202/Y204, T185/Y187), GSK-3α/β (S21/S9), HSP27 (S78/S82), and STAT2 (Y689), while it downregulated the energy regulator AMPKα1 to a quarter of its level compared to the control. KIM-161 upregulated the activity of checkpoint kinase 2 (Chk-2), a kinase that responds to cellular stress in HL60 (a p53-negative cancer cell) (Wolf and Rotter, 1985; van Jaarsveld et al., 2020). In addition, it reduced the activities of cAMP response element binding protein (CREB), c-Jun NH_2_-terminal kinases (JNK1/2/3), and HSP60 to lesser degrees (36.7, 26.0, and 28.5%, respectively). Meanwhile, c-Jun was slightly enhanced (15.8%) under the effect of KIM-161 compared to the untreated cells.

#### Effect of KIM-161 on Tubulin Polymerization

**FIGURE 9:**
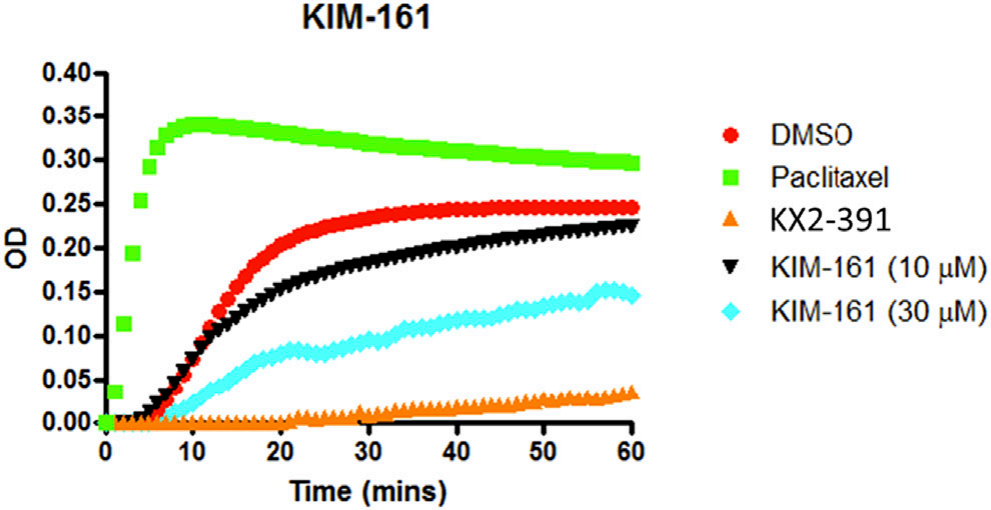
Tubulin polymerization assay for KIM-161 at 10 and 30 µM compared to paclitaxel (10 µM) and KX2-391 (10 µM) as positive controls. The negative control (vehicle) contained DMSO (1% v/v). The effect on the tubulin assembly was monitored turbidometrically at 340 nm every 30 s for 60 min at 37°C. Fold inhibition of tubulin polymerization was calculated using the V_*max*_ value for each reaction. The results represent the mean for three separate experiments.

KX2-391 (KX-01), the prototype of KIM-161, is a known powerful tubulin polymerization inhibitor. Consequently, we investigated its effect on tubulin polymerization using a cell-free assay that also included paclitaxel as another positive reference that affects tubulin function through stabilization of tubulin polymers (Figure 9). Obviously, KIM-161 showed much weaker tubulin polymerization inhibition than KX2-391 at 10 and 30 µM concentrations (Figure 9). This indicates that the new scaffold produces its cytotoxic activities via mechanisms other than tubulin inhibition.

#### Changes in Cytotoxic Activity of KIM-161 in the Presence of *N*-Acetylcysteine

**FIGURE 10:**
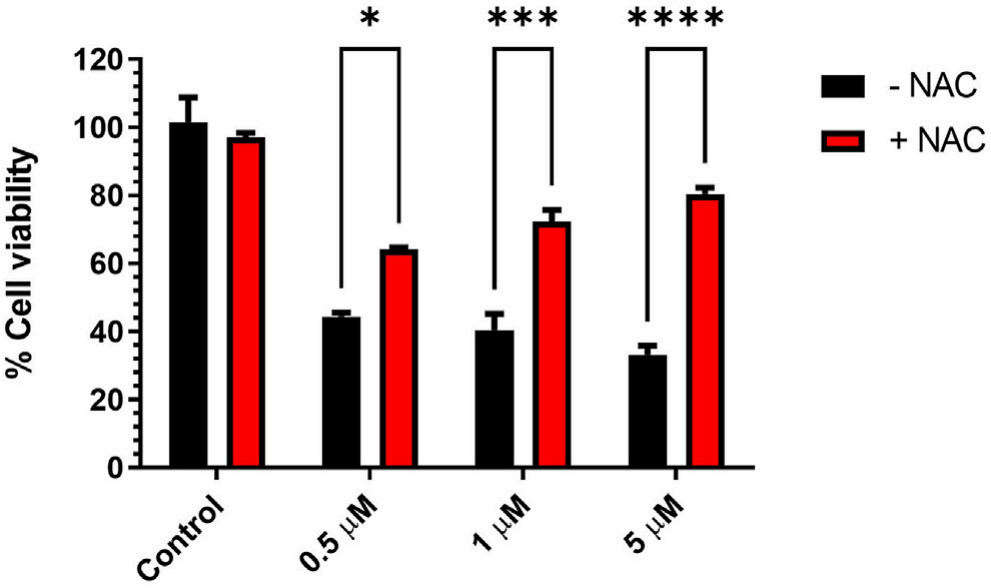
Effect of antioxidant pretreatment on viability of HCT116 treated with KIM-161. Cells were pretreated with NAC (5 mM) for 1 h, followed by KIM-161 at concentrations 0.5, 1.0, and 5.0 µM for 48 h. Cell viability was expressed as a percentage of vehicle control and was measured by the MTT assay (average of two independent experiments). Statistical significance was determined by two-way ANOVA using Šídák’s multiple comparisons test. * = *p* < 0.05; *** = *p* ≤ 0.001; **** = *p* < 0.0001.

Oxidative stress expressed as the reactive oxygen species (ROS) level is an important marker in cell death under the effect of certain cytotoxic drugs. Elevation of ROS may occur as a direct mechanism of some drugs such as Adriamycin (Kim et al., 2019) and *trans*-cinnamaldehyde (Afanas’ev, 2011; Ka et al., 2003) (safe covalent natural compound) or indirectly by the modulation of certain cellular contents (Teppo et al., 2017; Perillo et al., 2020). The involvement of ROS elevation and oxidative stress in the cytotoxic activities of KIM-161 on HCT116 was assessed. In this assay, the antioxidant *N*-acetylcysteine (NAC) was added at a very high concentration (5 mM) to check if it reverses the cytotoxic activities of KIM-161 on HCT116. At low KIM-161 concentration (0.5 µM), the cytotoxic effect was slightly altered by NAC addition compared to the control (no NAC added). However, increasing the concentration of KIM-161 caused a higher involvement of this factor in the anticancer effect (Figure 10).

#### Physicochemical Profiling of KIM-161

In silico measurements showed that KIM-161 has a calculated logP (cLogP) of 3.39 and a topological polar surface area (TPSA) of 71.0, which were obtained using the Marvin calculator plugin available in MarvinSketch from ChemAxon (Budapest, Hungary). The logP calculator employs a variation on the atomic fragment method originally described by Viswanadhan et al. (1989). The TPSA calculator uses the fragment summation method described by Ertl et al. (2000).

KIM-161 was also screened for its *in vitro* absorption/distribution/metabolism/elimination properties in a battery of high-throughput plate-based assays. The results are as follows: maximum kinetic aqueous solubility in 2% DMSO/phosphate-buffered saline (PBS), 19.9 µM; stability in mouse liver microsomes (MLM), 14.8 min; stability in human liver microsomes (HLM), 22.9 min; percentage of KIM-161 remaining in MLM in the presence and in the absence of nicotinamide adenine dinucleotide phosphate (NADPH) were unchanged after 60 min; plasma protein binding—percent free of the drug in mouse plasma protein at 37°C, 5%; stability in mouse plasma at 37°C, >300 min. In addition, the compound did not inhibit activity of human cytochrome P450 (CYP450) 3A4, 2D6, and 2C9 (IC_50_ values ≥ 10.0 µM). The data are summarized in Table 3.

**TABLE 3:** Physicochemical and pharmacokinetic properties of KIM-161.

Properties	KIM-161
CLogP	3.39
TPSA	71.0
Max solubility in 2% DMSO/PBS	19.9 µM
MLM stability	14.8 min
HLM stability	22.9 min
% Remaining in MLM with NADPH	Unchanged >60 min
% Remaining in MLM without NADPH	Unchanged >60 min
Mouse plasma protein binding (% free at 37°C)	5%
Stability in mouse plasma at 37°C	>300 min
CYP450 inhibition (IC_50_)	
3A4	>10.0 µM
2D6	>10.0 µM
2C9	>10.0 µM
Reactivity with GSH	No reaction

Because KIM-161 contains α,β-enone moiety, it was investigated for its reactivity with glutathione (GSH), an important cellular component for maintaining the desired redox balance (Aquilano et al., 2014). Monitoring the reaction with LC/MS assured that KIM-161 could not react with GSH as it remained intact after incubation with GSH at 37°C for 48 h (Supplementary Section S2).

## Discussion

The replacement of the outer ring phenyl of the 4-biphenylacetamide scaffold (KX chemotype) with 4-benzyliden-5(4*H*)-imidazolone provided compounds with good activities against cancer cell lines, especially the compound KIM-161. To consider as significant viable lead, it was fundamental to see signs of optimizable profile of KIM-161 corroborated by clear SAR features. In this regard, the presence of small methyl group was tolerated while bulkier phenyl weakened the activity to less than one-tenth (KIM-161 vs. **4g**, Table 1). The core imidazole-5-one ring was necessary because the open analog (**5**) of **4c** (KIM-161) was 20–40 times less active on MCF7 and PC3 cells.

There is some evidence that the cytotoxic activities correlated with higher electron density on the benzylidene moiety. For instance, the *p*-chloro analog **4b** (KIM-121) inhibited breast cancer and prostate cancer cell lines at significantly lower potencies than the *p*-methoxy (**4c**, KIM-161), the *p*-hydroxy-*m*-methoxy **4d**, and the unsubstituted **4a**. However, a confirmation of this SAR feature needs a larger set of compounds.

The physicochemical profiling of KIM-161 provided reassuring information about the drug-likeness of this lead (Table 3). Its good solubility (~20 μg/ml) was more than its IC_50_ values and concentrations used in MoA testing. Another advantage of this compound was its tameness with CYP450 metabolizing enzymes since modulators of this system are prone to drug–drug interactions. KIM-161 contains a carboxamide group that is potentially at risk for enzymatic hydrolysis, the “control” liver microsomal studies offer an indication of whether or not these molecules may be hydrolyzed in the liver. The compound demonstrated moderate stability in MLM, and there was no indication of liability to hydrolysis as indicated by the results of the NADPH-absent experiment. The compound also showed no significant difference between mouse and human microsomal stability, but it may need some optimization in the future to increase its half-life if it should go to further development. The stability in plasma is complimentary to the MLM test as a predictor for liability of hydrolysis. This test confirmed that KIM-161 is stable toward hydrolytic enzymes. The plasma protein binding of this compound (5%) lies in the range of many drugs currently in the market. All of these profiling tests boosted KIM-161 as a viable lead for the new type of anticancer compounds.

Based on the chemical scaffold, KIM-161 was supposed to inhibit primarily the Src family kinases. However, we failed to find any Src family kinases in the list of the top 20 kinases that were inhibited by KIM-161 even after using two different kinase profiling tests (PamChip® and Human Phospho-Kinase Array®; Table 2 and Figure 8, respectively). The two tests revealed a broad-range inhibition of multiple tyrosine kinases by KIM-161, and this is well aligned with previous reports associated with the preestablished Src family kinase inhibitors. For instance, dasatinib and bosutinib, two known Src inhibitors, affected several other kinases in various cancer models (Remsing Rix et al., 2009; Li et al., 2010; Liu et al., 2010). Likewise, our investigations define a set of signaling nodes modulated by KIM-161 that can be considered targets in multiple hematological cancer types and possibly in other epithelial cancer types. It is not a surprise to find that KIM-161 strongly inhibited a large set of tyrosine kinases, including multiple members of the BRK family kinases (SRM and FRK), ITK kinase (a TEC family member), FLT family kinase like VEGFRs, and JAK family kinase (JAK1).

It is highly important to note that KIM-161 targets the matrix of identified tyrosine kinase family members beyond the Src kinases in HL60 cells, pointing toward its utility in various cancer subtypes like breast cancer, ovarian cancer, and other hematological cancers those express the identified target tyrosine kinases of KIM-161 in the active state (Miah et al., 2014, 2019; Goel et al., 2018; McClendon and Miller, 2020).

Another interesting outcome to ponder from our study is to find the importance of KIM-161 target kinases like the BRK family kinases in various hematological cancer types. How these kinases influence cancer biology, treatment sensitivity, and overall prognosis remains to be understood. Given the role of genetic alterations in tyrosine kinases and sensitivity to tyrosine kinase inhibition, it should be useful to examine these KIM-161 tyrosine kinase targets for evidence of genetic alterations. It is also possible that KIM-161 targets play important roles in signal transduction in leukemia cancer cells that are addicted to upstream tyrosine kinases such as JAK, BTK, MYC, etc., which can be targeted with KIM-161. These upstream tyrosine kinases are known to signal through Src kinases and may require the function of other KIM-161 kinase targets for signal transduction. Thus, our study suggests combination strategies of KIM-161 with other YK inhibitors or signal transduction inhibitors that attack critical signaling pathways important for tumor maintenance.

In general, our findings suggest that KIM-161 could simultaneously inhibit signaling at multiple nodes originating from various tumor microenvironment cues. The direct target(s) of this compound has not been fully understood in as much as tirbanibulin is still revealing its polypharmacology nature (Wang et al., 2021). Our work with KIM-161 proved that changes in the KX2-391 skeleton changes the cytotoxic profile from dual Src/tubulin inhibition to other targets.

## Materials and Methods

### Chemical Synthesis

All melting points were uncorrected and measured using the capillary melting point instrument BI 9100 (Barnstead Electrothermal, UK). ^1^H-NMR spectra were determined on an AVANCE-III 600 MHz and AVANCE-III HD 850 MHz spectrometers (Bruker, Germany), and chemical shifts were expressed as parts per million against TMS as an internal reference (King Fahd Center for Medical Research and Faculty of Science, King Abdulaziz University, Jeddah, Saudi Arabia). LC/MS analyses were performed on an Agilent 6320 Ion Trap HPLC–ESI-MS/DAD (Santa Clara, CA, USA) with the following settings: the analytes were separated using an Macherey-Nagel Nucleodur C18 column (150 mm length × 4.6 mm i.d., 5 µm) (Macherey-Nagel GMBH and Co. KG, Duren, Germany). Mobile-phase was the isocratic elution using a mixture of acetonitrile and 0.01 formic acid in water (80: 20, v/v). The flow rate was 0.4 ml/min, and the total run time was 20 min. Purities were reported according to the percentage of peak areas at a wavelength of 280 nm. HRMS was performed in LTQ-XL Linear Ion Trap Mass Spectrometer coupled with Accela autosampler and Accela pump (Thermo Fisher Scientific Inc., San Jose, CA, USA). The ion source was the electrospray ionization compartment. The system was controlled with Xcalibur® Thermo Fisher Scientific Inc., ver. 2.07 SP1. The spay voltage was 5.0 kv; sheath gas flow rate, 45 ml/min; auxiliary gas, 10 ml/ min; sweep gas, 5 ml/ min; capillary voltage, 60 v; capillary temperature, 320°C; and scan range, +100–700 *m*/*z*. The collision energy was 35 V; the column used was Eclipse Plus C18, 3.5 μm, 4.6 × 100 mm (Agilent, Palo Alto, USA); the column oven was set at 25°C. The tray temperature was 20°C. The mobile system was composed of (A) acetonitrile and (B) water containing 5 mM ammonium acetate, and 1 ml glacial acetic acid, 100%. The flow rate was 400 μL/ min. The injection volume was 5 μL. The pump was programed to deliver 65% A: 35% B. The data were confirmed with the aid of NIST 2017, using MS interpreter software version BETA 3.1A build January 05, 2017. Column chromatography was performed on silica gel 60 (particle size, 0.06–0.20 mm).

#### General Procedure for Preparation of (*Z*)-4-arylidene-2-substituted oxazol-5(4*H*)-one intermediates 2a-h

The acylglycine **1a** or **1b** (10 mmol) was mixed with an equimolar amount of appropriate aldehyde, acetic anhydride (1.9 ml, 2 equiv.), and freshly dried sodium acetate (0.08 g, 0.1 equiv.). The mixture was heated at 80°C for 30 min, then cooled. The resulting solid was washed with water, then with sodium bicarbonate solution, and dried by vacuum filtration. The yellow solid was washed several times with petroleum ether and was used without further purification for the next step.

#### General Procedure for Synthesis of 4a-h

The appropriate oxazolone **2a-h** (2 mmol) was mixed with amine **3** (0.48 g, 2 mmol) in dry pyridine (6 ml) under inert atmosphere and heated to 100°C for 8 h in the STEM Integrity 10™ Reactor (Cole Parmer, England). The mixture was cooled and poured into the ice/HCl mixture. The precipitated solid was collected by filtration, washed with water, and purified by silica gel chromatography (gradient petroleum ether to 10% ethyl acetate in petroleum ether).

##### (*Z*)-*N*-Benzyl-2-(4-(4-benzylidene-2-methyl-5-oxo-4,5-dihydro-1*H*-imidazol-1-yl)phenyl)acetamide **4a**

This compound was off-white solid (yield 33%), mp 164–165°C. ^1^H-NMR (850 MHz, DMSO-*d*_6_) δ 8.6 (t, 1H, J = 6.0 Hz), 8.2 (d, 2H, J = 7.3 Hz), 7.4 (t, 2H, J = 7.6 Hz), 7.4–7.5 (m, 3H), 7.3 (d, 2H, J = 8.0 Hz), 7.3 (t, 2H, J = 7.5 Hz), 7.2–7.3 (m, 3H), 7.1 (s, 1H), 4.3 (d, 2H, J = 6.0 Hz), 3.5 (s, 2H), 2.23 (s, 3H). ^13^C-NMR (214 MHz, DMSO-*d*_6_) δ 170.2, 169.6, 163.4, 139.8, 138.8, 137.5, 134.5, 132.4, 132.2, 130.6, 130.5, 129.2, 128.8, 127.9, 127.7, 127.3, 126.1, 42.7, 42.3, 16.7. LC-MS (ESI), RT = 3.30 min; *m/z* 410.0 [M + H]^+^.

##### (*Z*)-*N*-Benzyl-2-(4-(4-(4-chlorobenzylidene)-2-methyl-5-oxo-4,5-dihydro-1*H*-imidazol-1-yl)phenyl)acetamide **4b**

This compound was off-white solid (yield 47%), mp 209–211°C. ^1^H-NMR (850 MHz, DMSO-*d*_6_) δ 8.6 (t, 1H, J = 5.8 Hz), 8.3–8.3 (d, 2H, J = 8.6 Hz), 7.6–7.6 (d, 2H, J = 8.6 Hz), 7.4–7.5 (d, 2H, J = 8.3 Hz), 7.3–7.4 (d, 2H, J = 8.3 Hz), 7.3 (t, 2H, J = 7.4 Hz), 7.2–7.3 (m, 3H), 7.1 (s, 1H), 4.3 (d, 2H, J = 6.0 Hz), 3.5 (s, 2H), 2.2 (s, 3H). ^13^C-NMR (214 MHz, DMSO-*d*_6_) δ 170.2, 169.5, 164.0, 139.8, 139.2, 137.5, 135.2, 134.0, 133.5, 132.1, 130.5, 129.4, 128.8, 127.9, 127.7, 127.3, 124.5, 42.7, 42.3, 16.7. LC-MS (ESI), RT = 4.6–5.1 min; *m/z* 443.9 [M + H]^+^.

##### (*Z*)-*N*-Benzyl-2-(4-(4-(4-methoxybenzylidene)-2-methyl-5-oxo-4,5-dihydro-1*H*-imidazol-1-yl)phenyl)acetamide **4c**

This compound was off-white solid (yield 45%), mp 185°C. ^1^H-NMR (850 MHz, CDCl_3_) δ 8.1 (d, 2H, J = 8.6 Hz), 7.3 (d, 2H, J = 8.0 Hz), 7.2–7.3 (m, 2H), 7.2 (d, 1H, J = 7.5 Hz), 7.1 (s, 1H), 7.1 (t, 4H, J = 7.4 Hz), 6.9 (d, 2H, J = 8.8 Hz), 4.3 (d, 2H, J = 6.0 Hz), 3.8 (s, 3H), 3.5 (s, 2H), 2.2 (br s, 3H). ^13^C-NMR (214 MHz, CDCl_3_) δ 170.0, 138.0, 135.8, 134.4, 130.8, 128.8, 127.8, 127.7, 127.6, 114.5, 55.5, 43.8, 43.3, 16.3. LC-MS (ESI), RT = 3.30 min; *m*/*z* 440 [M + H]^+^. HRMS (ESI), RT = 4.25 min, *m/z* 440.63712 [M + H]^+^, formula C_27_H_25_N_3_O_3_.

##### (*Z*)-*N*-Benzyl-2-(4-(4-(4-hydroxy-3-methoxybenzylidene)-2-methyl-5-oxo-4,5-dihydro-1*H*-imidazol-1-yl)phenyl)acetamide **4d**

This compound was off-white solid (yield 28%), mp 199–200°C. ^1^H-NMR (850 MHz, CDCl_3_) δ 8.0 (s, 1H), 7.5 (d, 1H, J = 7.3 Hz), 7.4 (d, 2H, J = 7.8 Hz), 7.3 (t, 2H, J = 7.6 Hz), 7.2 (d, 1H, J = 7.8 Hz), 7.2 (t, 4H, J = 8.0 Hz), 7.1 (s, 1H), 6.9 (d, 1H, J = 8.3 Hz), 6.0 (br s, 6H), 5.8 (br s, 6H), 4.4 (d, 2H, J = 6.2 Hz), 3.9 (s, 3H), 3.6 (s, 2H), 2.2 (s, 3H). ^13^C-NMR (214 MHz, CDCl_3_) δ 170.0, 169.9, 160.0, 148.3, 146.7, 138.0, 136.1, 135.5, 132.8, 130.7, 128.8, 127.7, 127.6, 126.9, 114.7, 113.8, 77.2, 56.0, 43.8, 43.3, 16.6. LC-MS (ESI), RT = 3.06 min; *m*/*z* 456.4 [M + H]^+^. HRMS (ESI), RT = 3.22 min, *m/z* 456.13508 [M + H]^+^, formula C_27_H_25_N_3_O_4_.

##### (*Z*)-*N*-Benzyl-2-(4-(4-benzylidene-5-oxo-2-phenyl-4,5-dihydro-1*H*-imidazol-1-yl)phenyl)acetamide **4e**

This compound was yellow solid (yield 54%), mp 218–219°C. ^1^H-NMR (850 MHz, DMSO-*d*_6_) δ 8.6–8.6 (t, 1H), 8.4–8.4 (d, 2H, J = 8.6 Hz), 7.6–7.6 (d, 2H, J = 8.6 Hz), 7.5 (d, 2H, J = 7.7 Hz), 7.5 (t, 1H, J = 7.4 Hz), 7.4 (t, 2H, J = 7.8 Hz), 7.3 (d, 2H, J = 8.3 Hz), 7.3–7.3 (m, 3H), 7.2–7.3 (m, 5H), 4.2 (d, 2H, J = 5.7 Hz), 3.5 (s, 2H). ^13^C-NMR (214 MHz, DMSO-*d*_6_) δ 170.2, 161.8, 139.8, 139.3, 137.3, 135.6, 134.3, 133.5, 133.2, 132.1, 130.4, 129.5, 129.5, 129.0, 128.9, 128.8, 128.1, 127.7, 127.3, 126.5, 42.7, 42.3. LC-MS (ESI), RT = 3.46 min; *m/z* 469.3 [M-3]^+^.

##### (*Z*)-*N*-Benzyl-2-(4-(4-(4-chlorobenzylidene)-5-oxo-2-phenyl-4,5-dihydro-1*H*-imidazol-1-yl)phenyl)acetamide **4f**

This compound was pale yellow solid (yield 21%), mp 218–220°C. ^1^H-NMR (850 MHz, DMSO-*d*_6_) δ 8.6–8.6 (m, 1H), 8.4–8.4 (m, 2H, J = 8.6 Hz), 7.6–7.6 (m, 2H, J = 8.6 Hz), 7.5 (d, 2H, J = 7.7 Hz), 7.5 (t, 1H, J = 7.4 Hz), 7.4 (t, 2H, J = 7.8 Hz), 7.3 (d, 2H, J = 8.3 Hz), 7.3–7.3 (m, 3H), 7.2–7.3 (m, 5H), 4.29 (d, 2H, J = 5.7 Hz), 3.5 (s, 2H). ^13^C-NMR (214 MHz, DMSO-*d*_6_) δ 170.2, 161.8, 139.8, 139.3, 137.3, 135.6, 134.3, 133.5, 133.2, 132.1, 130.4, 129.5, 129.5, 129.0, 128.9, 128.8, 128.1, 127.7, 127.3, 126.5, 42.7, 42.3. LC-MS (ESI), RT = 6.10 min; *m/z* 507.0 [M + H]^+^.

##### (*Z*)-*N*-Benzyl-2-(4-(4-(4-methoxybenzylidene)-5-oxo-2-phenyl-4,5-dihydro-1*H*-imidazol-1-yl)phenyl)acetamide **4g**

This compound was pale orange-yellow solid (yield 32.4%), mp 193°C. ^1^H-NMR (850 MHz, DMSO-*d*_6_) δ 8.6–8.6 (t, 1H), 8.4–8.4 (d, 2H, J = 8.6 Hz), 7.6–7.6 (d, 2H, J = 8.6 Hz), 7.55 (d, 2H, J = 7.7 Hz), 7.52 (t, 1H, J = 7.4 Hz), 7.40 (t, 2H, J = 7.8 Hz), 7.37 (d, 2H, J = 8.3 Hz), 7.3–7.3 (m, 3H), 7.2–7.3 (m, 5H), 4.29 (d, 2H, J = 5.7 Hz), 3.55 (s, 2H). ^13^C-NMR (214 MHz, DMSO-*d*_6_) δ 170.2, 161.8, 139.8, 139.3, 137.3, 135.6, 134.3, 133.5, 133.2, 132.1, 130.4, 129.5, 129.5, 129.0, 128.9, 128.8, 128.1, 127.7, 127.3, 126.5, 42.7, 42.3. LC-MS (ESI), RT = 5.60 min; *m/z* 502.0 [M + H]^+^.

##### (*Z*)-*N*-Benzyl-2-(4-(4-(4-hydroxy-3-methoxybenzylidene)-5-oxo-2-phenyl-4,5-dihydro-1*H*-imidazol-1-yl)phenyl)acetamide **4h**

This compound was pale orange-yellow solid (yield 38%), mp 109–112°C. ^1^H-NMR (850 MHz, DMSO-*d*_6_) δ 9.95 (s, 1H), 8.6–8.6 (t, 1H), 8.27 (d, 1H, J = 1.6 Hz), 7.98 (dd, 1H, J = 1.6, 8.3 Hz), 7.56 (dd, 1H, J = 1.2, 8.2 Hz), 7.5–7.5 (m, 1H), 7.5–7.5 (m, 1H), 7.5–7.5 (m, 1H), 7.51 (br s, 1H), 7.50 (d, 1H, J = 7.1 Hz), 7.5–7.5 (t, 1H), 7.4–7.4 (m, 4H), 7.3–7.3 (m, 3H), 7.2–7.3 (m, 5H), 7.2–7.2 (m, 1H), 6.91 (d, 1H, J = 8.0 Hz), 4.29 (d, 2H, J = 6.0 Hz), 3.87 (s, 3H), 3.5–3.6 (d, 2H). ^13^C-NMR (214 MHz, DMSO-*d*_6_) δ 170.3, 170.2, 170.2, 168.9, 161.3, 159.1, 151.3, 148.2, 141.5, 139.8, 138.8, 137.3, 133.6, 133.5, 133.3, 132.0, 131.6, 130.4, 130.4, 129.4, 129.3, 129.2, 129.1, 128.9, 128.9, 128.8, 128.1, 128.0, 127.7, 127.4, 127.3, 125.9, 123.8, 116.5, 116.3, 56.3, 56.0, 42.7, 42.3. LC-MS (ESI), RT = 3.2 min; *m/z* 518.0 [M + H]^+^.

#### Procedure for Synthesis of (*E*)-2-acetamido-*N*-(4-(2-(benzylamino)-2-oxoethyl)phenyl)-3-(4-methoxyphenyl) acrylamide (5)

The oxazolone **2c** (2 mmol, 0.434 g) was mixed with 2-(4-aminophenyl)-*N*-benzylacetamide **3** (2.2 mmol, 0.529 g) in acetonitrile (6 ml) under inert atmosphere and heated to 80°C for 8 h in STEM Integrity 10™ Reactor (Cole Parmer, England). The mixture was cooled and poured into ice/HCl mixture. The precipitated solid was collected by filtration, washed with water, and purified by silica gel chromatography (gradient dichloromethane to 5% MeOH in dichloromethane). The product **5** was white solid (yield 25%), mp 189–200°C. ^1^H-NMR (850 MHz, DMSO-*d*_6_) δ 9.85 (s, 1H), 9.49 (s, 1H), 8.50 (t, 1H, J = 6.0 Hz), 7.63 (d, 2H, J = 8.3 Hz), 7.6–7.6 (m, 2H, J = 8.8 Hz), 7.31 (t, 2H, J = 7.6 Hz), 7.2–7.3 (m, 5H), 7.0–7.0 (m, 2H, J = 8.8 Hz), 6.97 (s, 1H), 4.27 (d, 2H, J = 6.0 Hz), 3.80 (s, 3H), 3.44 (s, 2H), 2.04 (s, 3H). ^13^C-NMR (214 MHz, DMSO-*d*_6_) δ 170.7, 170.0, 164.7, 160.0, 140.0, 138.2, 131.7, 131.6, 129.5, 129.2, 128.7, 127.7, 127.6, 127.2, 127.1, 120.5, 114.5, 55.7, 42.7, 42.3, 23.3. LC-MS (ESI), RT = 2.30 min; *m/z* 457.9 [M + H]^+^.

### Methods for Biological Experiments on Solid Tumor Cell Lines

All cell lines were purchased from commercial cell line banks. All biological work involving human cell lines and animal derived material were approved by the institutional ethical committee at the Center of Excellence in Genomic Medicine Research (approval code: 13-CEGMR-Bioeth-2021). All experiments were performed in triplicate, and the IC_50_ values were calculated considering all results.

#### Cell Culture

MCF7 (breast), PC3 (prostate), and HCT116 (colon) cancer cell lines were obtained from ATCC, Manassas, VA, USA. Cells were grown in Roswell Park Memorial Institute-1640 (RPMI-1640) medium (Thermo Fisher Scientific, Inc., Waltham, MA, USA), supplemented with 10% heat inactivated fetal bovine serum (FBS), 50 units/mL of penicillin, and 50 mg/ml of streptomycin and maintained at 37°C in a humidified atmosphere containing 5% CO_2_. The cells were maintained as “monolayer culture” by serial subculturing.

#### SRB Cytotoxicity Assay

Cytotoxicity was determined using the sulforhodamine B (SRB) method as previously described by Skehan et al. (1990). Exponentially growing MCF7 and PC3 cells were collected using 0.25% Trypsin-EDTA and were then seeded in 96-well plates at a density of 1,000–2,000 cells/well in 10% FBS and penicillin/streptomycin supplemented RPMI-1640 medium. After 24 h, the cells were treated with different concentrations of the test compounds and incubated for 72 h at 37°C in a humidified atmosphere containing 5% CO_2_. Next, the cells were fixed by trichloroacetic acid (TCA) (10% final concentration in wells) directly to the wells and incubated for 1 h at 4°C, then the cells were washed with distilled water five times to remove excess TCA, media, serum proteins, and metabolites, followed by air drying. After that, the fixed cells were stained with 0.4% SRB dissolved in 1% acetic acid for 10 min at room temperature, and excess stain was removed by quickly rinsing with 1% acetic acid. The residual acetic acid was removed completely by air drying the plates for 24 h, and the dye was solubilized with Tris buffer (pH = 7.4, 10 mM) for 5 min on a shaker at 1,600 rpm. The optical density of each well was measured spectrophotometrically at 564 nm with an ELISA microplate reader (ChroMate-4300, FL, USA). The IC_50_ values were calculated according to the equation for Boltzman sigmoidal concentration–response curve using the nonlinear regression fitting models (GraphPad®, Prism Version 5).

#### MTT Antiproliferative Assay

The test compounds were evaluated for antiproliferative activity using the MTT viability assay against the HCT116 cell line. The cells were seeded in triplicate in 96-well plates at a density of 10 × 10^3^ cells/mL in a total volume of 200 µL per well. 0.1% of DMSO was used as a vehicle control. Each well was treated with 2 µL of test compound, which had been preprepared as stock solutions in ethanol to furnish the concentration range of the study, 0.1–50 μM, and re-incubated for a further 72 h at 37°C in a humidified atmosphere containing 5% CO_2_. The culture medium was then removed, and the cells were washed with 100 µL of PBS, and 100 µL of MTT was added, to reach a final concentration of 1 mg/ml of MTT. The cells were incubated for 3 h in darkness at 37°C. After removal of MTT solution, solubilization was initiated through the addition of 200 ml of DMSO, and the cells were maintained at room temperature in darkness for 20 min to ensure thorough color diffusion before reading the absorbance with a microplate reader at 570 nm. The results were expressed as percentage viability relative to vehicle control (100%). The dose–response curves were plotted, and the IC_50_ values (concentration of drug resulting in 50% reduction in cell survival) were obtained using the commercial software package Prism (GraphPad® Software, Inc., La Jolla, CA, USA). All the experiments were repeated in at least three independent experiments.

### Methods for Biological Experiments on Leukemia Cell Lines

All cell lines were purchased from commercial cell line banks. All biological work involving human cell lines and animal derived material were approved by the institutional ethical committee at the Center of Excellence in Genomic Medicine Research (approval code: 13-CEGMR-Bioeth-2021).

#### Cell Culture and Reagents

HL60, NB4, and KG1a were purchased from CLS Cell Line Service GmbH (Eppelheim, Germany). All cell lines were cultured in RPMI-1640 medium supplemented with 10% FBS (Thermo Fisher Scientific) and ciprofloxacin (10 μg/ml; Cipla Limited; Mumbai, India), at 37°C with 5% CO_2_ in a humidified incubator.

#### Cell Viability Assay

The CellTiter-Blue® Cell Viability assay was acquired from Promega Corporation (Madison, WI, USA). The cell viability assay was performed as follows: the cells (10^4^/well) were incubated with a concentration gradient of the test compounds ranging from 0.01 to 100 μM, in 96-well plates for 48 h at 37°C. Subsequently, 20 µL of the CellTiter-Blue® Cell Viability reagent was added to each well and incubated for an additional 2 h for the development of fluorescence. The fluorescence emission was measured at 590 nm using the SpectraMax® i3x Multi-Mode Microplate Reader (Molecular Devices, LLC; San Jose, CA, USA.) and plotted against the drug concentrations to determine the IC_50_ of the test compounds.

#### Cell Cycle Analysis

The cells (3.5 × 10^5^/well) were incubated with 1 µM **4c (KIM-161)** at 37°C for 48 h. Subsequently, the cells were collected and washed twice with ice-cold PBS (1X). The washed cells were fixed on ice for 20 min using a fixation buffer containing paraformaldehyde. Hoechst 33342 (10 μg/ml; Thermo Fisher Scientific, Inc.) was used for staining. The cells were then incubated in the dark for 30 min on ice. A minimum total of 20,000 events were acquired using a BD FACSAria III flow cytometer. Flowlogic version 7.2.1 software (Inivai Technologies, Victoria, Australia) was used to obtain the percentages of cells in the G1, S, and G2/M phases in the singlet-gated population.

#### Apoptosis Detection by Caspase-3 Activity

FITC conjugated active caspase-3 antibody (BD biosciences, USA) was used to detect the active form of caspase-3 in the cells undergoing apoptosis. Cells were plated in a 6-well culture plate at a density of 0.5 × 10^6^ cells and harvested after 24 and 48 h of incubation with the test compound. The collected cells were washed twice with cold 1X PBS and then resuspended in 0.5 ml of BD Cytofix/Cytoperm solution followed by a 20-min incubation on ice. After incubation, the cells were washed twice in a BD Perm/Wash buffer (1X) and were labelled with 5 µL of FITC rabbit anti-caspase-3 antibodies. The labelled cells were washed again with wash buffer and resuspended in 0.5 ml of buffer and analyzed by acquiring a minimum of 5,000 events on the FACSAria III cell analyzer and sorter.

#### Tubulin Polymerization Assay

The assembly of purified bovine tubulin was monitored using a kit BK006 purchased from Cytoskeleton Inc. (Denver, CO, USA). The assay was carried out in accordance with the manufacturer's instructions in the tubulin polymerization assay kit manual using the standard assay conditions. The values reported represent the average values from two independent assays. Purified (>99%) bovine brain tubulin (3 mg/ml) in a buffer consisting of 80 mM PIPES (pH 6.9), 0.5 mM EGTA, 2 mM MgCl_2_, 1 mM GTP, and 10% glycerol was incubated at 37°C in the presence of either vehicle (2% v/v DMSO) or compound KIM-161 at concentrations of 10 and 30 µM in DMSO. KX2-391 and Paclitaxel were used as the positive controls (reference drugs). Light is scattered proportional to the concentration of the polymerized microtubules in the assay. Therefore, tubulin assembly was monitored turbidimetrically at 340 nm at 37°C in a Spectramax 340 PC spectrophotometer (Molecular Devices, Sunnyvale, CA, USA). The absorbance was measured at 30-s intervals for 60 min.

#### Cytotoxic Assay in Presence of Antioxidant *N*-Acetylcysteine

HCT116 cells were seeded at a density of 5 × 10^4^ cells/well in 96-well plates and left overnight to adhere. The cells were then pretreated with NAC (10 mM) for 1 h, followed by the addition of the test compound KIM-161, and incubation was continued for 48 h. Cell viability was expressed as a percentage of the vehicle control (1% v/v ethanol) and was assessed using the MTT assay (data are expressed as mean ± SEM). Statistical analysis was performed using two-way ANOVA.

#### PamChip® Tyrosine Kinase Microarray

Every compound had spiked into a leukemic (AML) cell line HL60 lysate at different concentrations (0.001, 0.01, 0.1, 1, 10, and 50 μM). In addition, DMSO had also spiked at a low concentration (2% v/v) to the HL60 lysate as the control. The samples were profiled on PamGene's tyrosine kinase (PTK) arrays in technical replicates, covering a total of 196 phosphorylation sites. The frozen leukemic HL60 cultured cell pellet was sent to PamGene. At PamGene, the cells were lysed in the presence of the lysis buffer and proteases inhibitor cocktail. The protein concentration was measured using the standard Bradford assay as per the PamGene SOPs. These lysates were further used for generating the PTK activity profiles. For spiking, KIM-161 was received in powder form and dissolved in DMSO at the PamGene facility, followed by storing it at −20°C in small aliquots. PamGene used its in-house bioinformatics toolbox in combination with other methods to generate the hypotheses of signaling pathways.

#### Human Phosphokinase Array Assay

The phosphorylation profile of 48 kinases was performed using the Proteome Profiler Human Phospho-Kinase Array an antibody array kit, by R&D Systems (Minneapolis, MN, USA). Total protein was extracted from the cell lysates treated with 365 nM KIM-161 for 10 h using the lysis buffer provided in the kit and quantified using the Bio-Rad DC Protein Assay kit (USA). The nitrocellulose membrane set (A&B), pre-spotted with the capture antibodies, was first blocked with 1 ml of Array buffer 1 at RT for 1 h in an 8-well multi-dish on a rocking platform shaker. Approximately 600 µg of protein concentrate (<334 µL) was diluted in the Array buffer 1 to a final volume of 2 ml. 1 ml of the protein sample was added onto the membrane set and incubated overnight at 4°C on a rocking platform shaker. Subsequently, membranes were washed with 15 ml of 1× wash buffer thrice for 5 min at RT, and the excess buffer was completely drained. The membranes A&B were then incubated with appropriate detection antibody cocktails A&B that were reconstituted using the 1× Array buffer 2/3 for 2 h at RT. After the incubation was complete, the membranes were washed with 15 ml of 1× wash buffer thrice for 5 min at RT, and the excess buffer was completely drained. Streptavidin-HRP (1:2000), diluted in 1× Array buffer 2/3, was applied onto the membranes and incubated for 30 min at RT on a rocking platform shaker. The membranes were washed thrice, and Chemi Reagent 1&2 were applied in equal volumes for detection on C-DiGit®Blot Scanner (LI-COR Odyssey Imaging Systems, Lincoln, Nebraska, USA) using the Image Studio 5.0 software.

### Physicochemical Property Assays

#### Maximum Kinetic Aqueous Solubility

Solubility assays were performed using the Millipore MultiScreen® HTS-PFC Filter Plates designed for the solubility assays (EMD Millipore, Billerica, MA). Assays were run in triplicate. The 96-well plates consist of two chambers separated by a filter. Liquid handling was performed using JANUS® Verispan and MTD workstations (Perkin Elmer, Waltham, MA). About 4 µL of the KIM-161 solution (10 mM in DMSO) was added to 196 µL of phosphate buffer (45 mM potassium phosphate, 45 mM sodium acetate, 45 mM ethanolamine, pH = 7.4) in the top chamber to give a final DMSO concentration of 2% and a theoretical drug concentration of 200 µM. The plates were gently shaken for 90 min and then subjected to vacuum. The insoluble drug was captured on the filter. 160 µL of the filtrate was transferred to 96-well Griener UV-Star® analysis plates (Sigma-Aldrich, St. Louis, MO) containing 40 µL of acetonitrile. The test compound concentration in the filtrate is measured by UV absorbance on a Spectromax® Plus microplate reader (Molecular Devices, Sunnyvale, CA) using SoftMax Pro Software v.5.4.5. The absorbances at five wavelengths (280, 300, 320, 340, and 360 nm) were summed to generate the UV signal. Standard curves were generated by adding 4 µL of five concentrations of the test compound in DMSO to 40 µL of acetonitrile in UV Star plates followed by 156 µL of the appropriate solubility medium. Analysis and statistics were performed using GraphPad® Prism v.5.04. The data were reported as the maximum concentration observed in the filtrate.

#### Stability in Mouse and Human Liver Microsomes

The clearance of KIM-161 in MLM or HLM was determined at 37°C as previously described (Yang et al., 2007). Both MLM and HLM were obtained from Merck KGaA (Darmstadt, Germany), Cat. No. M9441 (MLM) and M0317 (HLM). The assays were conducted in triplicate in 96-well deep polypropylene plates. KIM-161 was incubated with pooled liver microsomes from male CD-1 mice (Life Technologies, Grand Island, NY), tetrasodium NADPH and magnesium chloride for 60 min at 37°C with gentle shaking. At five time points, the reaction mixture aliquots were transferred to 96-shallow well stop plates on ice containing acetonitrile with 0.1 µM propafenone. The control reactions (lacking NADPH) were performed in a similar manner to demonstrate NADPH dependency of compound loss and assess the potential for hydrolysis of compounds in the liver tissue. The standard curves for the test compound were generated using five concentrations in triplicate that were processed as above but with zero incubation time. The stop plates were centrifuged at 2000*g* for 10 min, and then, the supernatant aliquots were transferred to a Waters Aquity® UPLC 700 µL 96-well sample plate with cap mat (Waters, Milford, MA). The amount of the compound remaining in the supernatant was quantified by LC/MS/MS using a Waters Xevo TQ MS (electrospray positive mode) coupled to a Waters Aquity® UPLC (BEH column, C18). Propafenone was used as the internal standard. GraphPad® Prism v.5.04 was used for nonlinear fitting of time course data to generate t_1/2_ values.

#### Inhibition Assay of Human CYP450 Enzymes

KIM-161 was assessed for its ability to inhibit the three major human CYP450 enzymes: 3A4, 2D6, and 2C9. The assays were run in triplicate. The expressed enzymes in insect Supersomes (Fisher Scientific, Waltham, MA) were used to minimize nonspecific binding and membrane partitioning issues (McMasters et al., 2007). The 3A4 assay uses testosterone as a substrate, and the analysis was performed by LC/MS/MS on a Waters Xevo TQ instrument as described above, using positive electrospray ionization. The assay acceptance criterion was 20% for all standards and 25% for the LLOQ. The 2D6 and 2C9 assays use fluorescent substrates (3-[2-(*N*,*N*-diethylamino)ethyl]-7-methoxy-4-methylcoumarin for 2D6; 7-methoxy-4-(trifluromethyl)coumarin for 2C9) and were analyzed on an Envision plate reader. GraphPad® Prism v.5.04 was used for nonlinear fitting of data to generate IC_50_ values.

#### Stability in Mouse Plasma

The assay was conducted at 37°C in triplicate. The test compound KIM-161 or procaine (the positive control) was tested at a final concentration of 1 µM in either 2.5% DMSO/CD-1 mouse plasma (Innovative Research, Novi, MI; sodium heparin added as anticoagulant; pH adjusted to 7.4 with 2N HCl on the day of use) or 2.5% DMSO/PBS (pH 7.4; 136.9 mM NaCl, 2.68 mM KCl, 8.1 mM Na_2_HPO_4_, 1.47 mM KH_2_PO_4_, 0.9 mM CaCl_2_, and 0.49 mM MgCl_2_). At seven time points, the reaction mixture aliquots were transferred to 96-shallow-well stop plates on ice containing acetonitrile with 0.1 µM propafenone. Samples were analyzed by LC/MS/MS on a Waters Xevo TQ instrument as described above using positive electrospray ionization. The assay acceptance criterion was 20% for all standards and 25% for the LLOQ. GraphPad® Prism v.5.04 was used for nonlinear fitting of data to generate t_1/2_ values.

#### Binding to Mouse Plasma Proteins

The equilibrium dialysis method for determining plasma protein binding was performed as previously described using 96-well dialyzer plates with a molecular weight cutoff of 5K (Harvard Apparatus, Holliston, MA) and a dual-plate rotator set to maximum speed (Harvard Apparatus, Holliston, MA) located in a 37°C incubator with a 10% CO_2_ atmospheric environment. The assays were run in triplicate. KIM-161 was added to CD-1 mouse plasma (Innovative Research, Novi, MI, sodium heparin added as anticoagulant; pH adjusted to 7.4 with 2N HCl on the day of use) in DMSO (final DMSO concentration 0.4%) to give a 10 µM final concentration. The drug/plasma mixture and buffer (Dulbecco's PBS 1X without calcium and magnesium, Mediatech, Inc., Herndon, VA) were placed in their respective sides; the wells were capped; and the plate was placed in the rotator and allowed to dialyze for 22 h. Following dialysis, aliquots of the buffer and plasma mixture were removed and mixed with aliquots of the opposite matrix in 96-well deep plates. The concentrations of the analytes from each side of the dialysis plate were determined by LC/MS/MS on a Waters Xevo TQ instrument as described above using positive electrospray ionization. The assay acceptance criterion was 20% for all standards and 25% for the LLOQ. The fraction unbound was calculated by dividing the drug concentration in the buffer side of the dialysis plate by the drug concentration in the plasma side.

#### Reactivity With GSH

GSH and solvents were purchased from Sigma-Aldrich. The LC/MS system is composed of an Agilent 1200 HPLC system, a solvent delivery module, a quaternary pump, an autosampler, and a column compartment (Agilent Technology, Germany). The column effluent was connected to an Agilent 6320 Ion Trap-ESI-MS. The column heater was set to 25 ± 2°C. The control of the HPLC system and data processing were performed using ChemStation (Rev. B.01.03 SR2-204) and 6300 Series Trap Control version 6.2 Build No. 62.24 (Bruker Daltonik GmbH). The analytes were separated using an Agilent Zorbax Extend-C18 column (80Å, 150 mm length × 4.6 mm, i.d., 5 μm) and Agilent-Zorbax Extend-C18 pre-column (Agilent Technologies, Palo Alto, CA, USA). The general MS adjustments were set as follows: capillary voltage, 4000 V; nebulizer, 35 psi; drying gas, 12 L/min; desolvation temperature, 350°C; ion charge control smart target, 150,000; and max. accumulation time, 150 ms. The Auto-MSn positive mode was applied. The mobile system was isocratic elution using 55% acetonitrile and 45% water containing 0.1% formic acid (w/v).

Freshly prepared 10 mM solution of GSH in water (50 µL) was mixed thoroughly with 1.9 ml Tris-citrate buffer (pH 7.5). To initiate the reaction, 50 µL of freshly prepared KIM-161 (10 mM) in DMSO was added and the mixture was stirred at 37°C. After the specified time, 0.4 ml of the reaction mixture was transferred to a vessel containing 40 µL phosphoric acid (10% in water) and mixed thoroughly, and diluted to 1 ml using acetonitrile. A volume of 1.5 µL was then injected for LC/MS analysis.

## Data Availability

The original contributions presented in the study are included in the Supplementary Material; further inquiries can be directed to the corresponding author.

## Abbreviations

ADME, absorption/distribution/metabolism/elimination; AMPKα1, AMP-activated protein kinase α1; BRK, breast tumor kinase; BTK, Bruton agammaglobulinemia tyrosine kinase; Chk-2, checkpoint kinase 2; CREB, cAMP response element binding protein; CYP 450, cytochrome P450; ERK1/2, extracellular signal-related kinase 1/2; FLT, FMS-like tyrosine kinase; FRK, FYN-related kinase; FYN, FYN oncogene related to SRC, FGR, YES; GSK-3α/β, glycogen synthase kinase 3α/β; HLM, human liver microsomes; HSP27, heat shock protein 27 kDa; ITK, IL2-inducible T-cell kinase; JAK1, Janus kinase 1; JNK, c-Jun NH_2_-terminal kinase; LCK, lymphocyte-specific protein tyrosine kinase; MLM, mouse liver microsomes; MYC, myelocytomatosis oncogene cellular homolog; NADPH, nicotinamide adenine dinucleotide phosphate; PBS, phosphate-buffered saline; SAR, structure–activity relationship; SRM, SRC-related kinase lacking C-terminal regulatory tyrosine and N-terminal myristoylation sites; STAT2, signal transducer and activator of transcription 2; TEC, tec protein tyrosine kinase; VEGFR, vascular endothelial growth factor receptor.
