# Supplementary material for: Insights on Cancer Cell Inhibition, Subcellular Activities, and Kinase Profile of Phenylacetamides Pending 1H-Imidazol-5-One Variants
Source: Front Pharmacol. 2022 Jan 5;12:794325. doi: 10.3389/fphar.2021.794325 (PMC8766756; doi:10.3389/fphar.2021.794325)
Supplement: Supplementary file 3 [file DataSheet1.docx]

Supplementary Material

# S1. Spectra of screened compounds


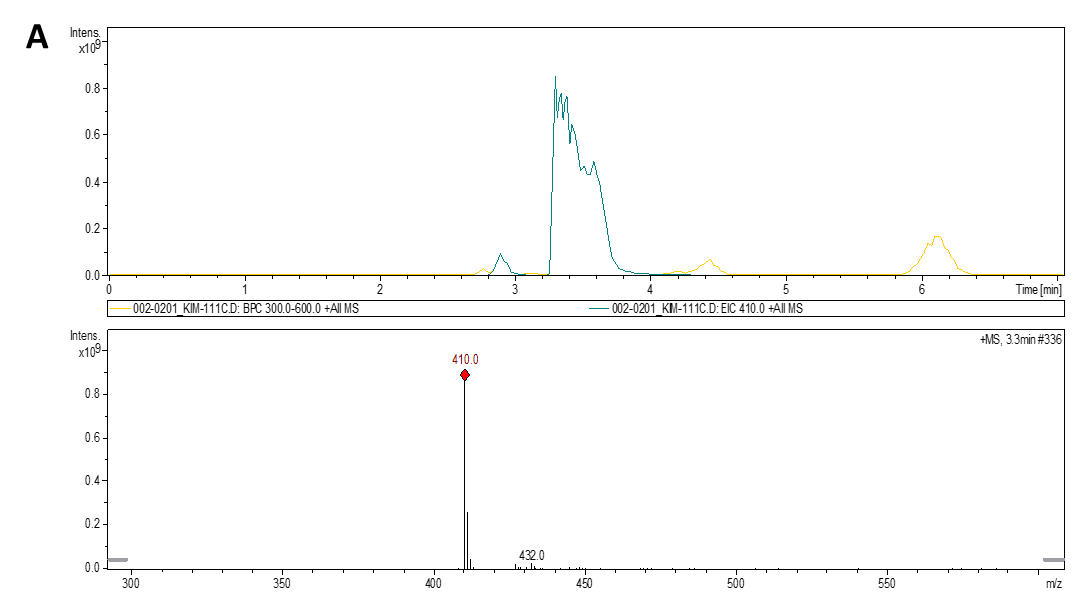


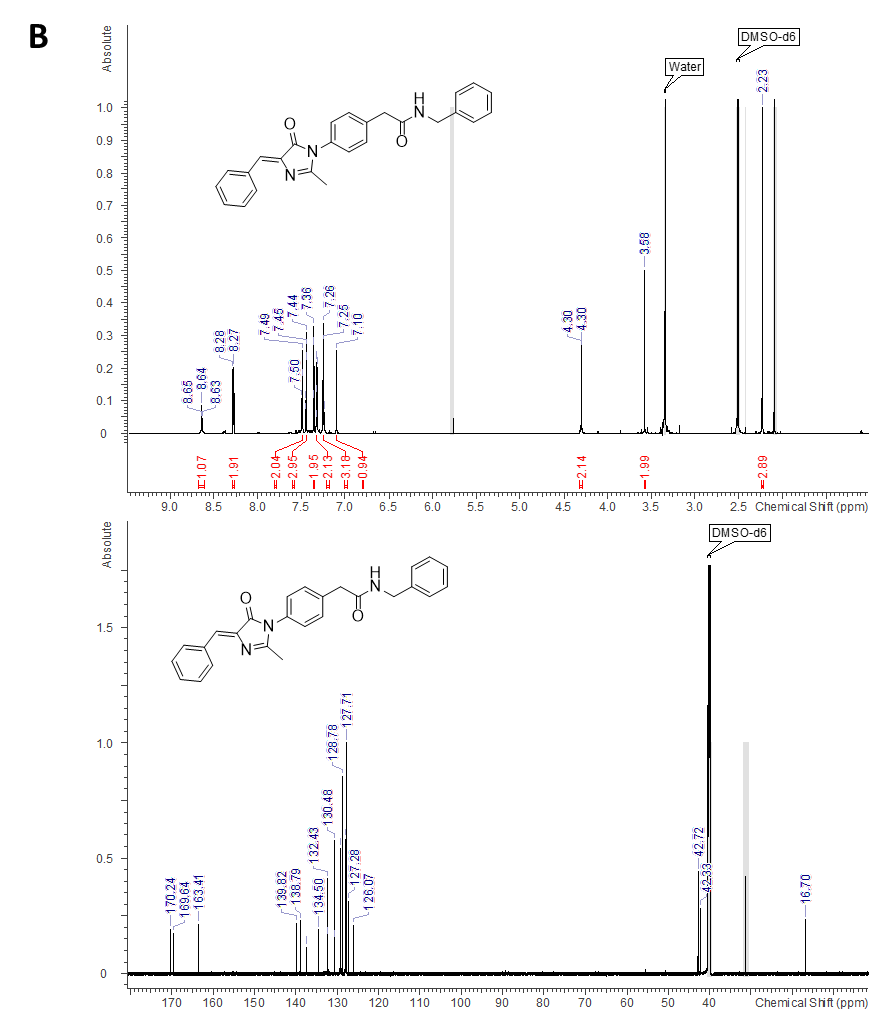


**Supplementary Figure 1.** Spectra of compound **4a**: (**A**) LCMS and (**B**) NMR


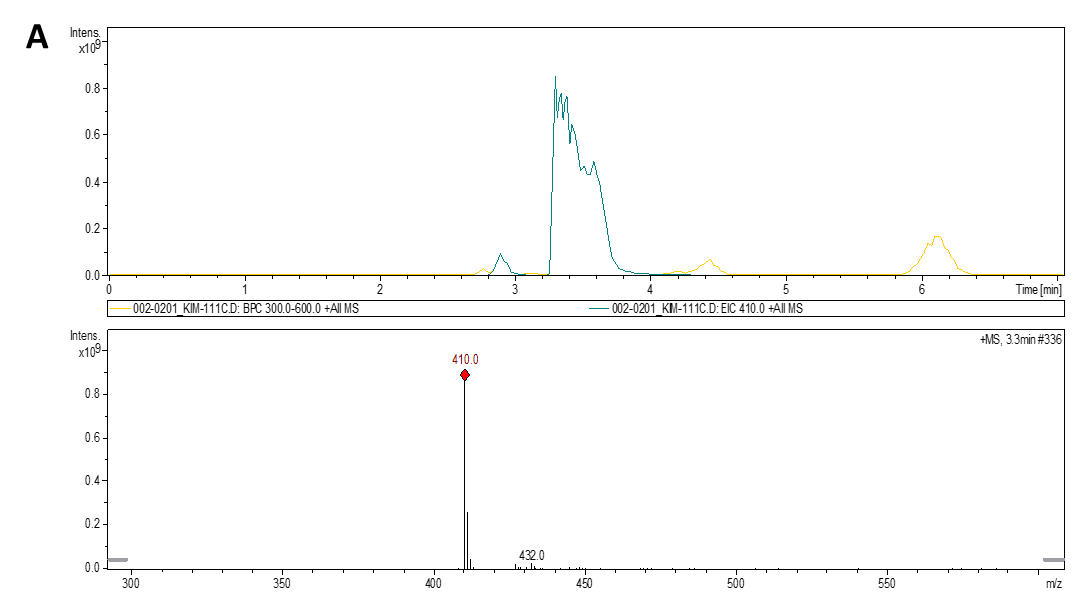


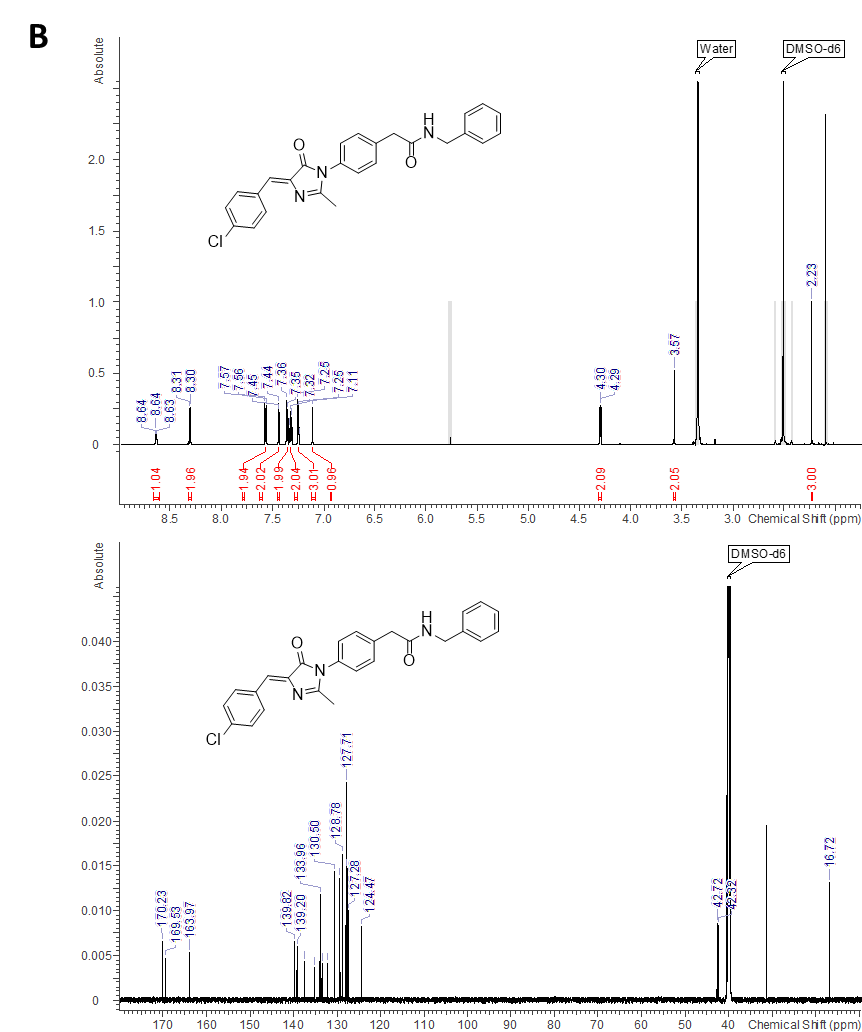


**Supplementary Figure 2.** Spectra of compound **4b**: (**A**) LCMS and (**B**) NMR


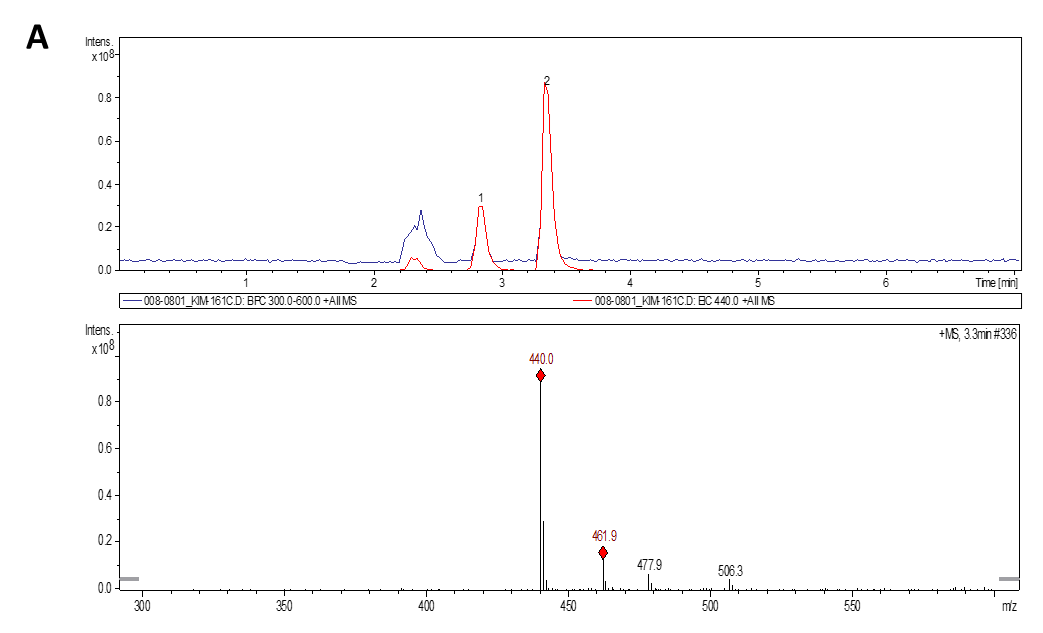


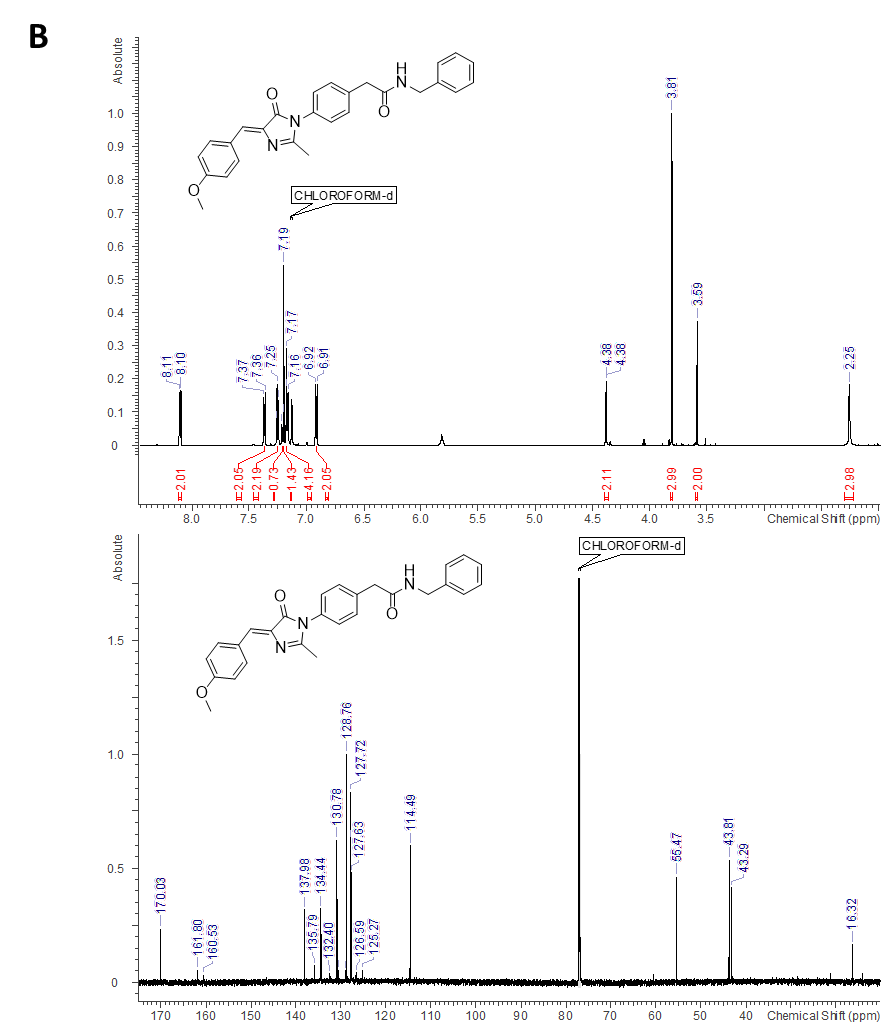


**Supplementary Figure 3.** Spectra of compound **4c**: (**A**) LCMS and (**B**) NMR


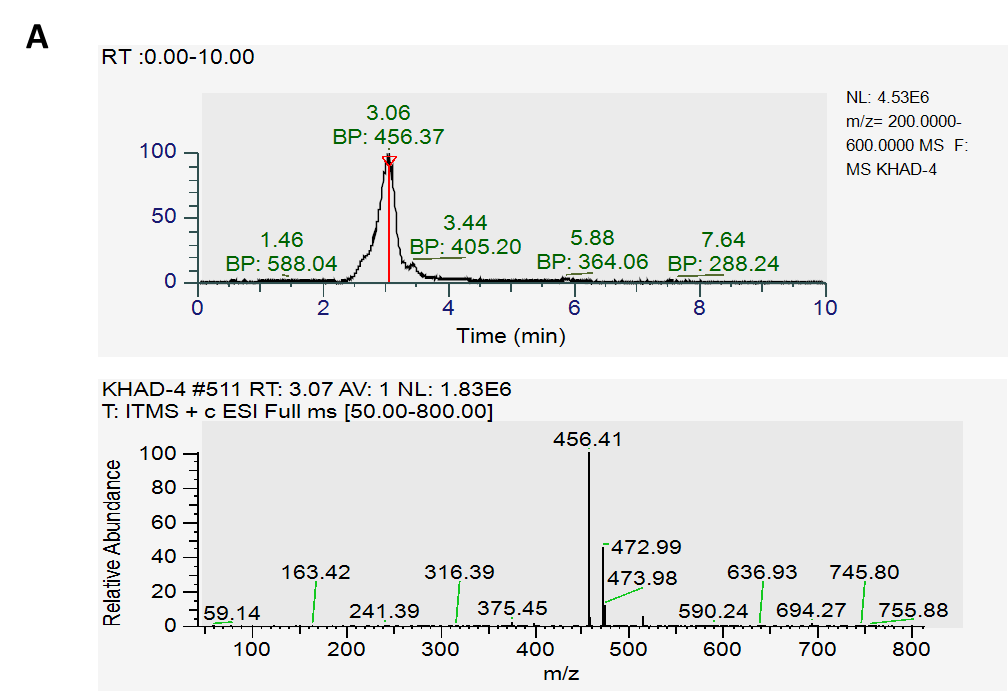


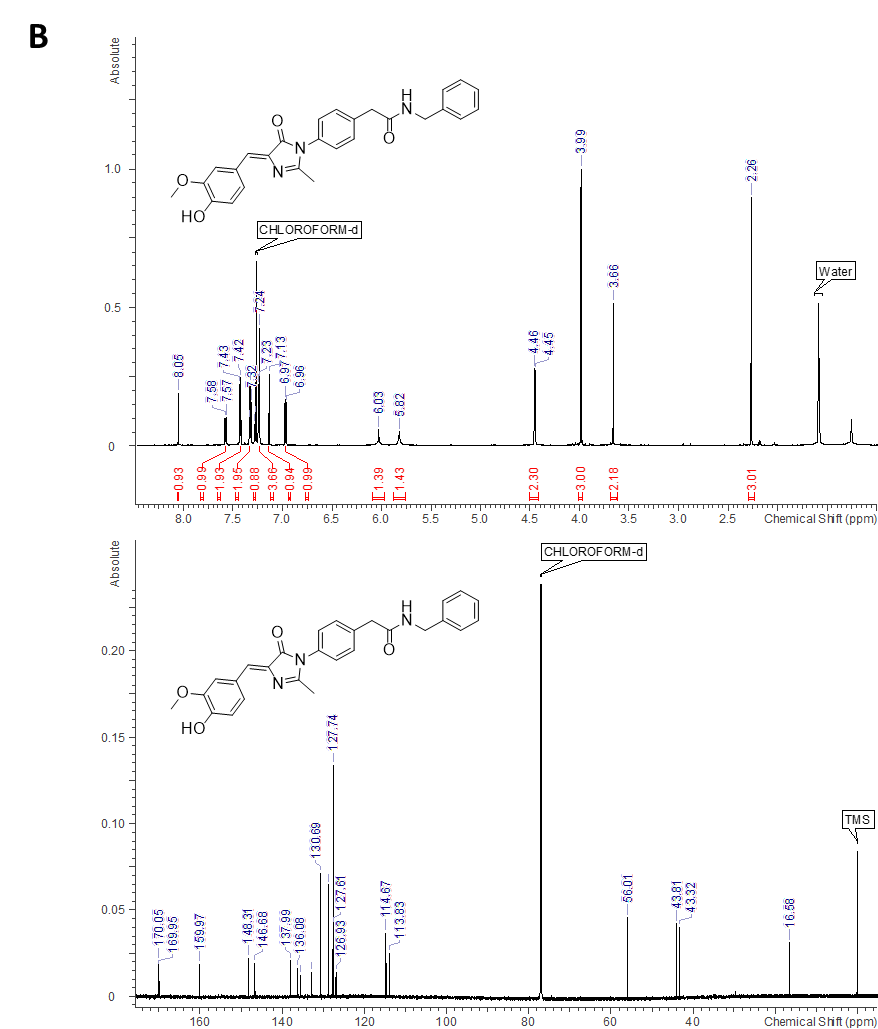


**Supplementary Figure 4.** Spectra of compound **4d**: (**A**) LCMS and (**B**) NMR


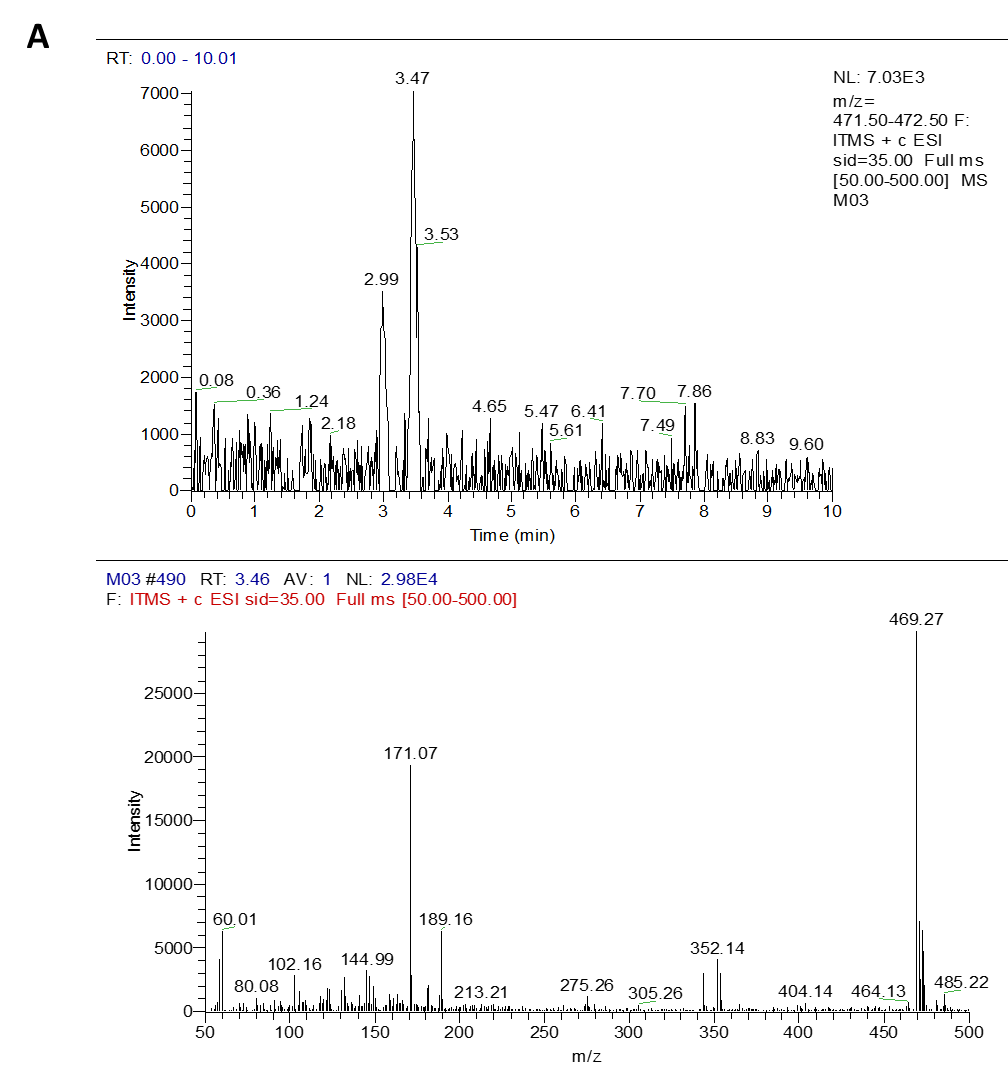


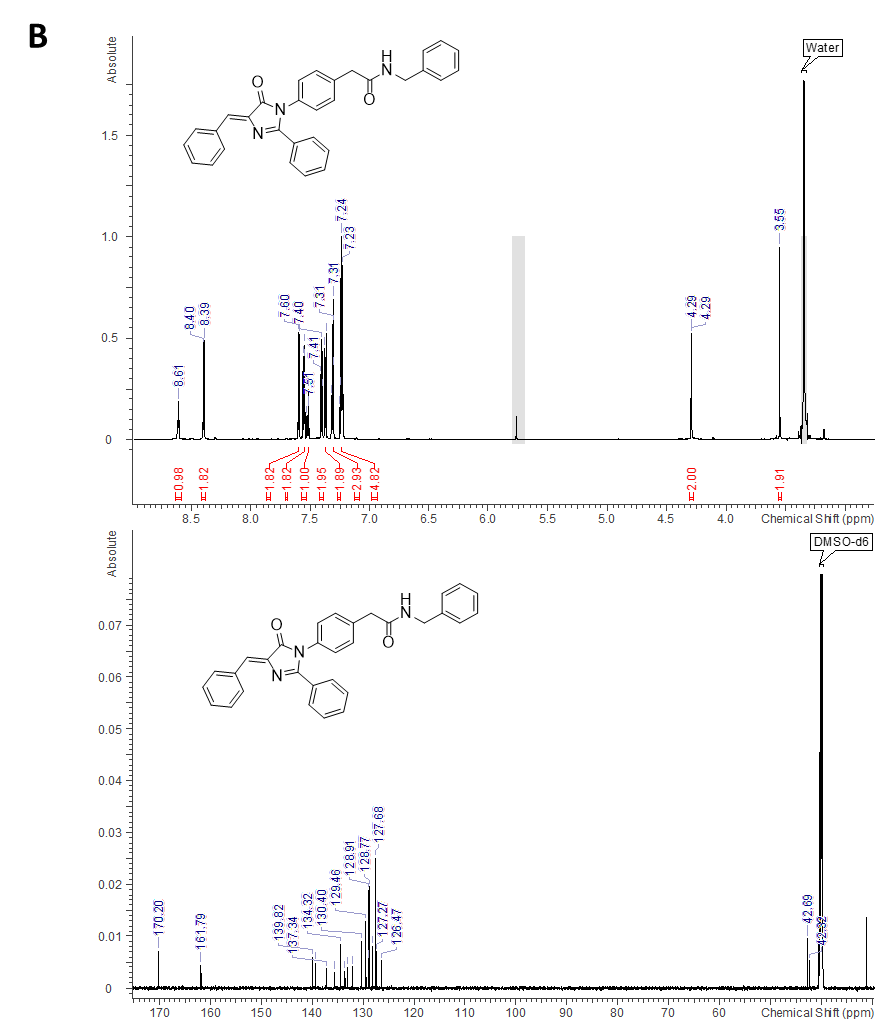


**Supplementary Figure 5.** Spectra of compound **4e**: (**A**) LCMS and (**B**) NMR


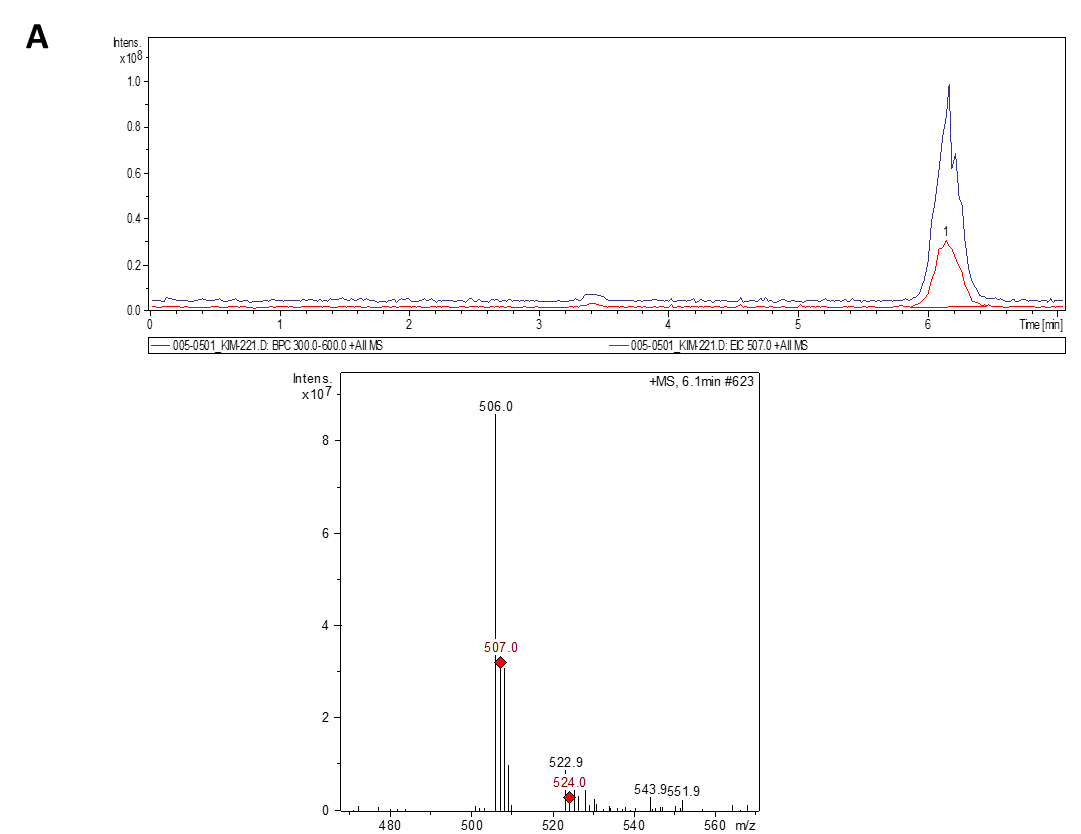


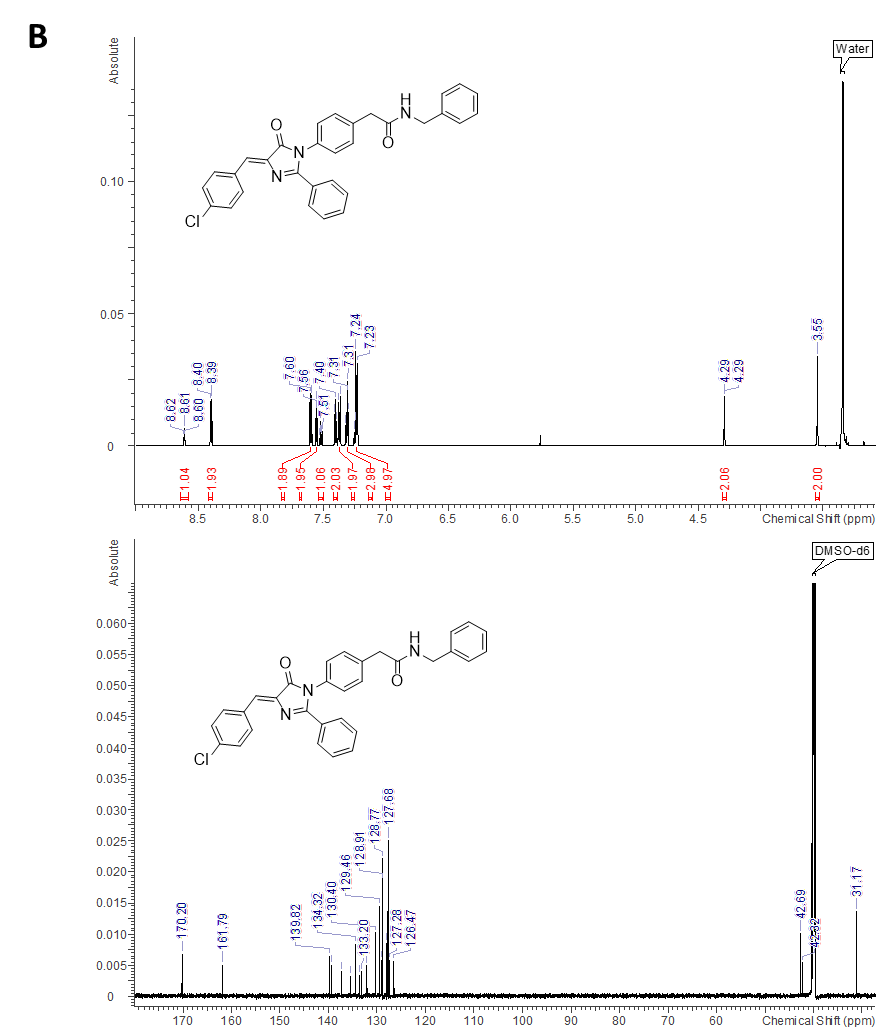


**Supplementary Figure 6.** Spectra of compound **4f**: (**A**) LCMS and (**B**) NMR


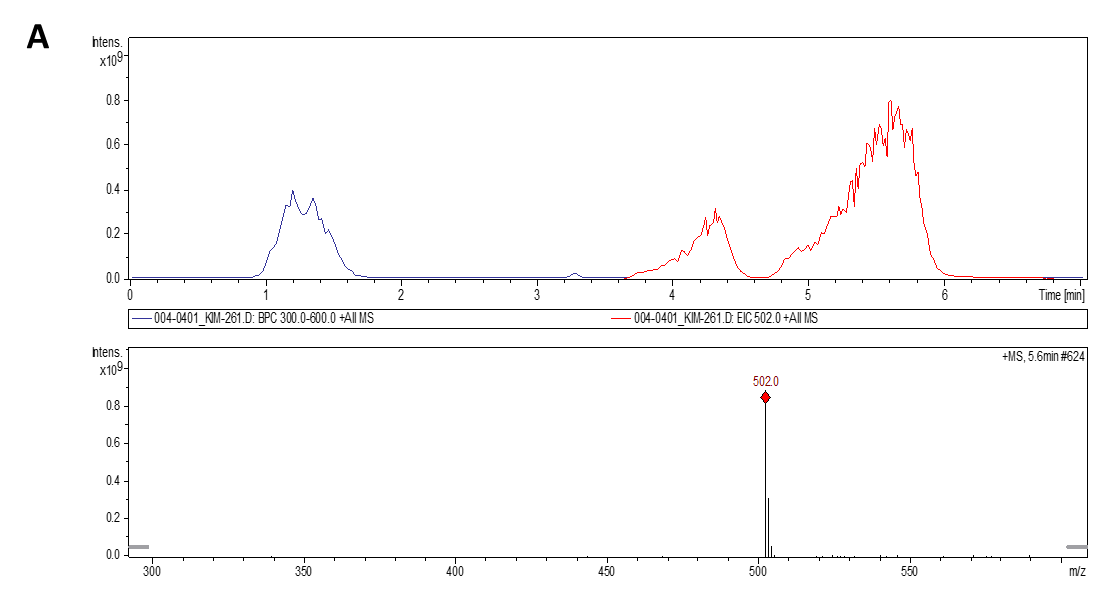


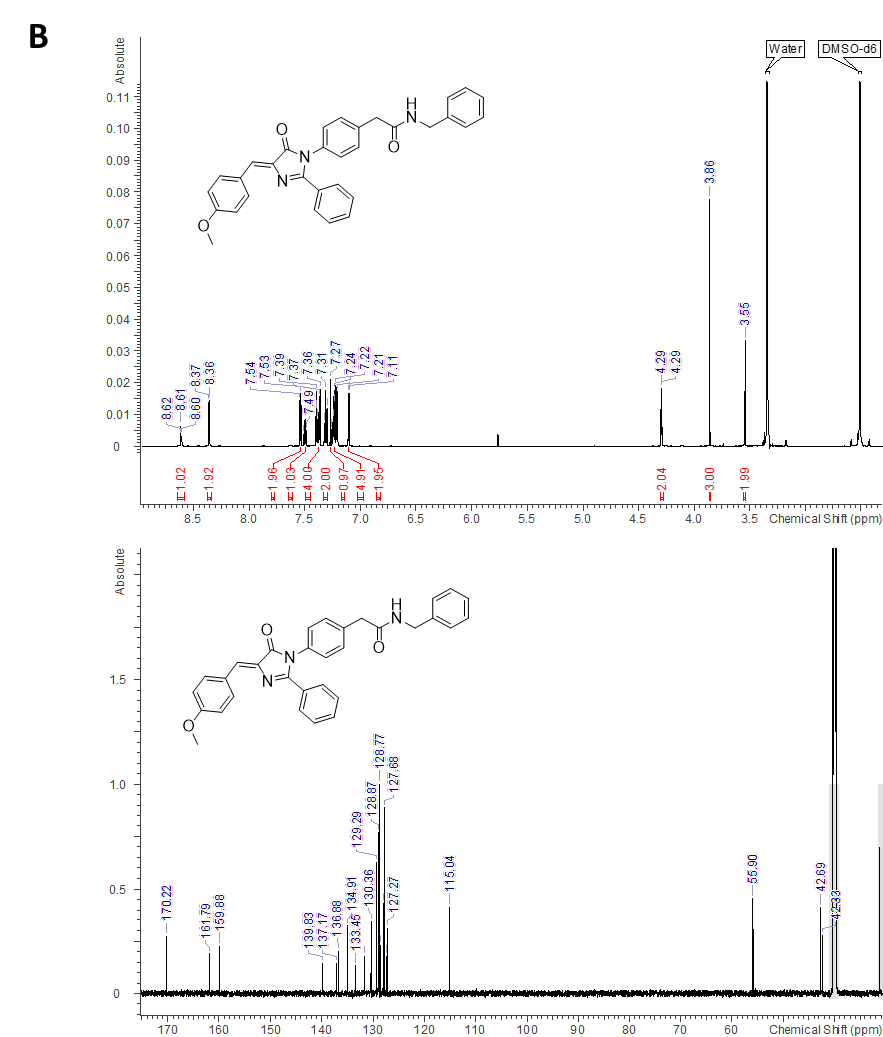


**Supplementary Figure 7.** Spectra of compound **4g**: (**A**) LCMS and (**B**) NMR


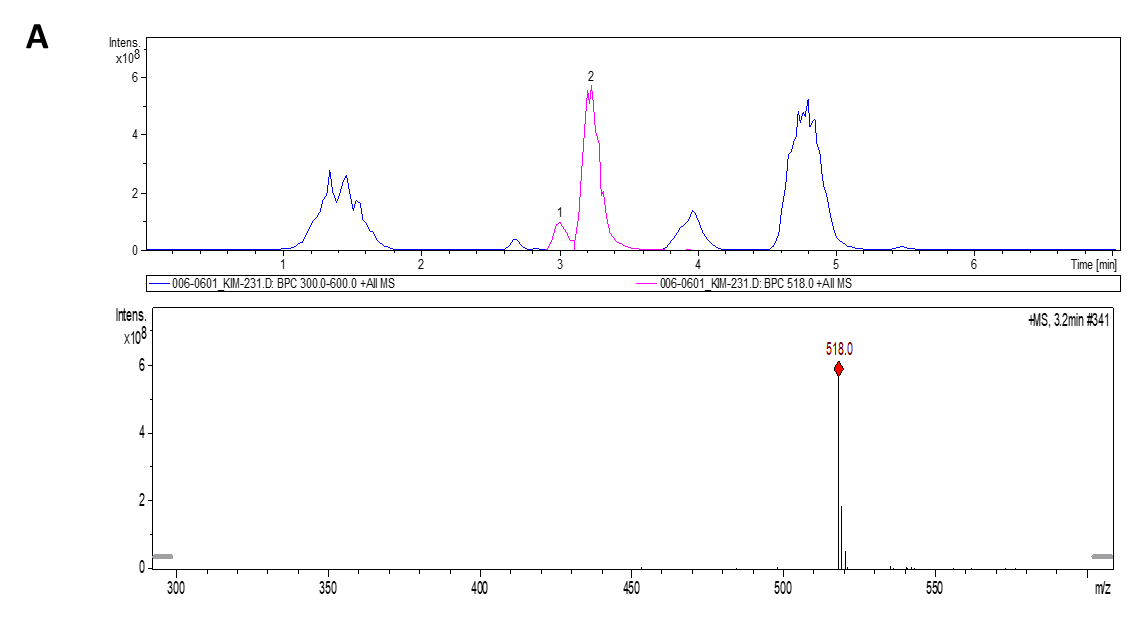


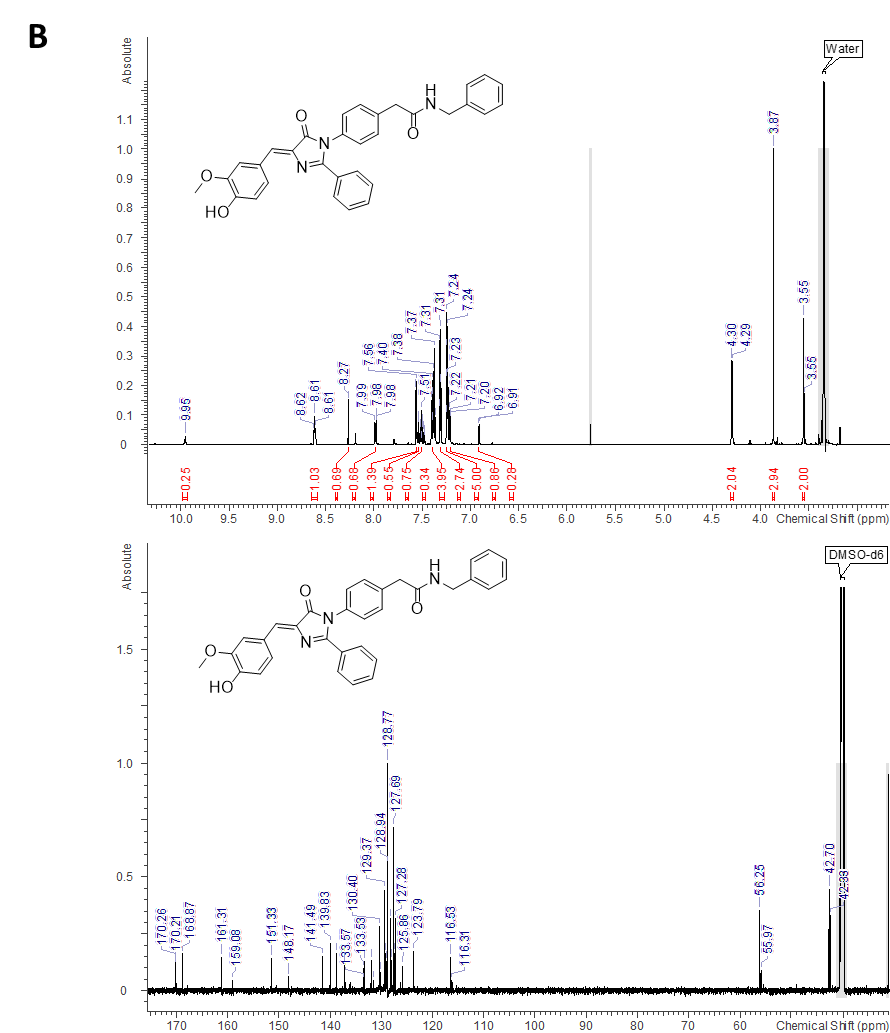


**Supplementary Figure 8.** Spectra of compound **4h**: (**A**) LCMS and (**B**) NMR


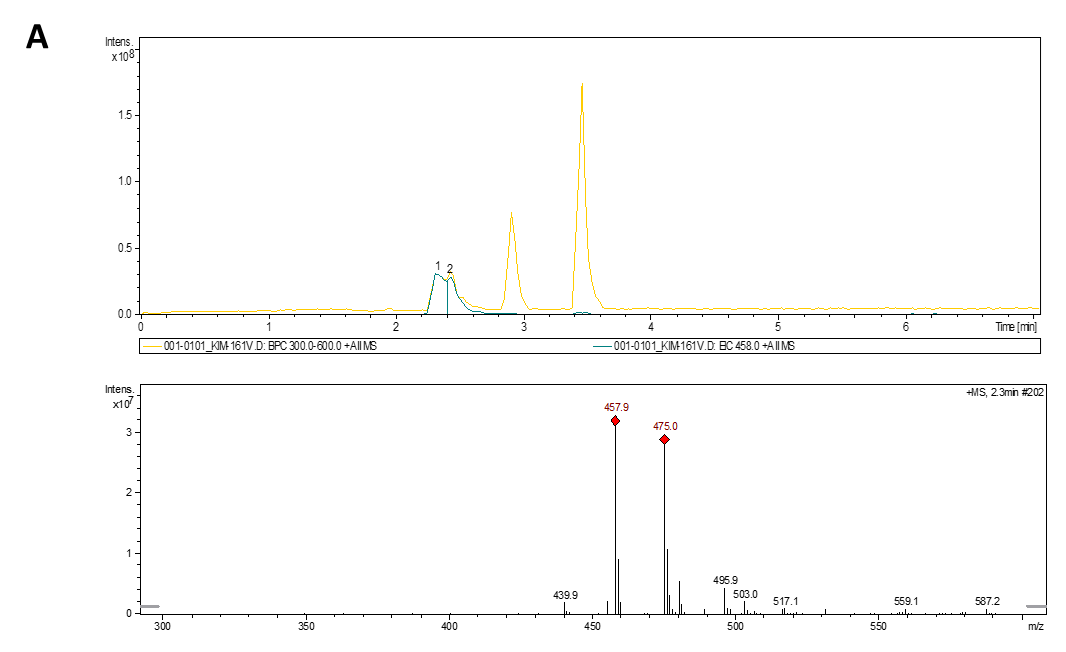


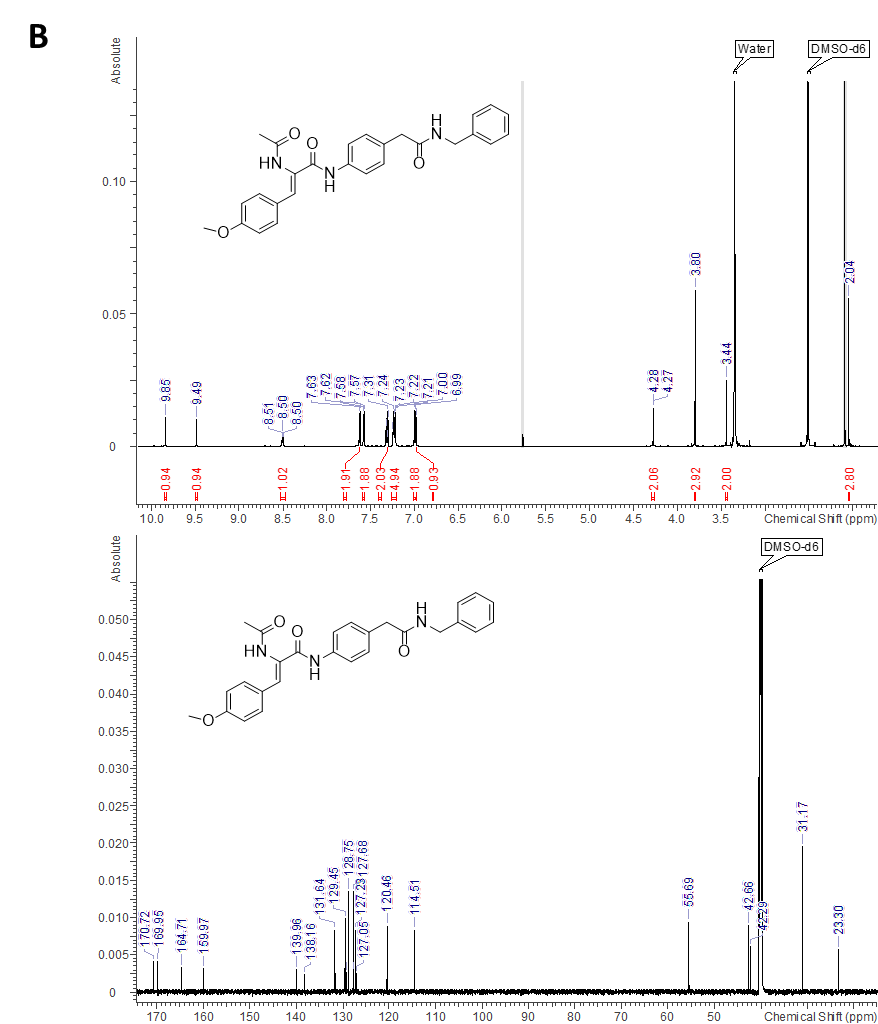


**Supplementary Figure 9.** Spectra of compound **5**: (**A**) LCMS and (**B**) NMR

# S2. Reactivity of KIM-161 with reduced glutathione

## Principle and Hypothesis

Reactivity of compounds with SH-containing cellular components is a problematic property that damage drug-likeness of the screened compound. It also questions the compound of being nuisance in assays and increase possibility of PAINS character (Baell and Nissink, 2018; Dahlin et al., 2021). Frequently, Michael Acceptors fall to this category because they usually react easily with the reduced glutathione (GSH) leading to non-specific cancer cytotoxic activities (Baell and Holloway, 2010; Baell and Walters, 2014; Tomašić and Mašič, 2012). Such compounds are characterized by being non-optimizable, non-progressible and show poor SAR.

Our compound (Z)-N-(4-(4-(4-methoxybenzylidene)-2-methyl-5-oxo-4,5-dihydro-1H-imidazol-1-yl)benzyl)-2-phenylacetamide (KIM) feature 4-alkylidene-4,5-dihydro-1H-imidazol-one nucleus that appear in PAINS databases. In addition, a similar scaffold was reported to make Michael Addition reaction with phenylthiols to for an adduct similar to A (Scheme 1) (Tikdari and Panah, 2005). We aimed to check if this potent compound reacts with GSH in vitro. Therefore, we adopted literature procedure that was used for this purpose (Jöst et al., 2014).

**Supplementary Scheme 1**. Possible reaction of GSH with KIM-161. Adduct product (A) results from Michael Addition reaction of GSH with KIM-161 Michael acceptor moiety highlighted in red. The product B may result from hydrolysis under condition of the reaction while product C may result from nucleophilic attack of the GSH on the carbonyl of the imidazolone ring.

## Instrumentation and Material

Glutathione (GSH), L-cysteine and solvents were purchased from Sigma-Aldrich. The LC-MS system is composed of an Agilent 1200 HPLC system, a solvent delivery module, a quaternary pump, an autosampler, and a column compartment (Agilent Technology, Germany). The column effluent was connected to an Agilent 6320 Ion Trap-ESI-MS. The column heater was set to 25 ±2 ◦C. The control of the HPLC system and data processing were performed using ChemStation (Rev. B.01.03 SR2-204) and 6300 Series Trap Control version 6.2 Build No. 62.24 (Bruker Daltonik GmbH). The analytes were separated using an Agilent Zorbax Extend-C18 column (80A˚, 150 mm length × 4.6 mm, i.d., 5 μm) an Agilent-Zorbax Extend-C18 pre-column (Agilent Technologies, Palo Alto, CA, USA). General MS adjustments were set as follows: capillary voltage, 4000 V; nebulizer, 35 psi; drying gas, 12 L/min; dessolvation temperature, 350 ◦C; ion charge control (ICC) smart target, 150,000; and max accumulation time, 150 ms. Auto-MSn positive mode was applied. Mobile Phase system: isocratic elution using 55% acetonitrile and 45% water containing 0.1% formic acid (w/v).

HPLC (UV) analysis was performed on Agilent 1200 series HPLC system (USA), equipped with Agilent 1200 pump (USA), Multiple wavelength Agilent 1200 detector; Agilent eclipse XDB-C-18 column (150×4.6 mm, 5 μm particle size). The mobile phase consisted of 0.1 trifluoracetic acid (TFA) in water (A) and 0.1 % TFA in acetonitrile (B). The mobile phase was applied in a gradient analysis using 90 A/ 10 B at zero time increased gradiently to 100% B after 7 min and kept till 12 min and returned back to 90 A/ 10 B at 15 min. Flow rate and sample volume were set to 1 ml/min and 20µl, respectively. The analysis was monitored at 254, 280 and 320 nm.

## Procedure:

Freshly prepared 10 mM solution of glutathione in water (50 µL) was mixed thoroughly with 1.9 mL TRIS-citrate buffer (pH 7.5). To initiate the reaction, 50 µL of freshly prepared tested compound (10 mM) in DMSO was added and the mixture was stirred at 37 °C. After specified time, 0.4 mL of the reaction mixture was transferred to a vessel containing 40 µL phosphoric acid (10% in water) and mixed thoroughly.

## Results

KIM-161 did not show any significant reaction with GSH even after mixing for 48 h. After 48, the UV spectrum showed >98% unreacted compound. The peaks that appear at RT 2.4 min (in the TIC spectrum only) is not related to any of the expected reaction product. The peak at 2.9 min showed the M+1 of KIM-161.


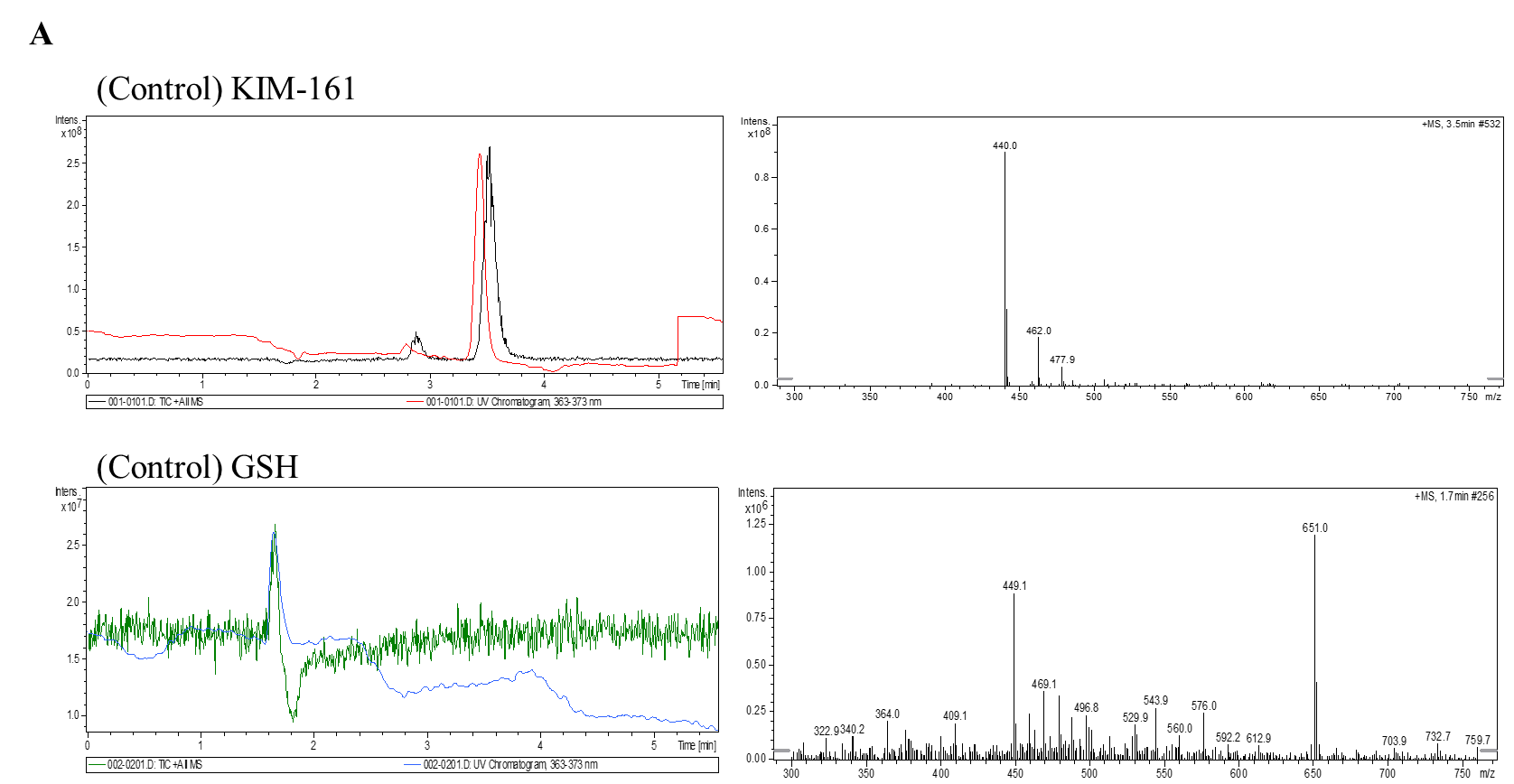


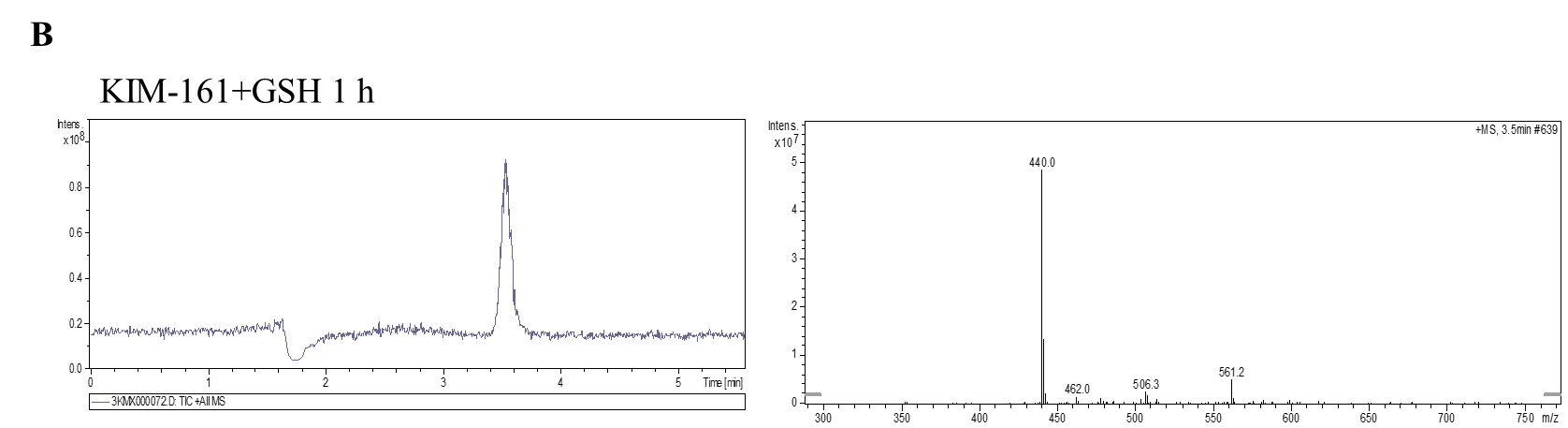


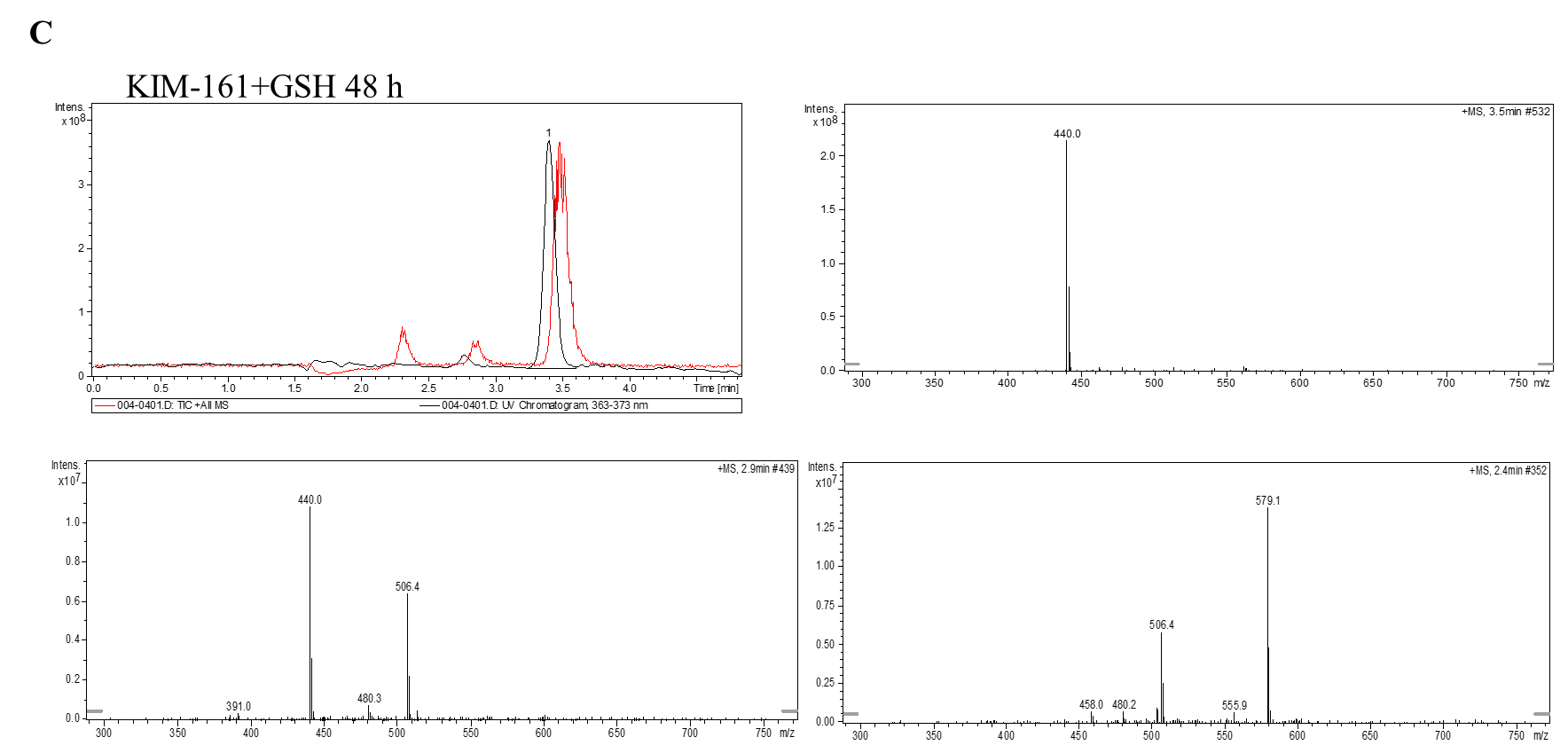


**Supplementary Figure 10**. Reactivity of KIM-161 to GSH. UV and LCMS spectra of KIM-161 and GSH alone (**A**). UV and LCMS spectra of KIM-161 after incubation with GSH for 1 h (**B**) and 48 h (**C**).

**Conclusion**

Summing up clear SAR, outstanding subcellular activities, and lack of reactivity towards GSH confirms that KIM-161 act by binding to specific target(s) similar to non-covalent inhibitors such as KX-01. However, we cannot confirm if this binding is followed by covalent interactions, an issue that lies within the scope of ongoing studies.

# References

Baell, J., and Holloway, G. (2010). New Substructure Filters for Removal of Pan Assay Interference Compounds (PAINS) from Screening Libraries and for Their Exclusion in Bioassays. J. Med. Chem., 53, 2719–2740. doi: 10.1021/JM901137J

Baell, J., and Nissink, W. (2018). Seven Year Itch: Pan-Assay Interference Compounds (PAINS) in 2017—Utility and Limitations. ACS Chem. Biol., 13, 36–44. doi: 10.1021/ACSCHEMBIO.7B00903

Baell, J., and Walters, M. A. (2014). Chemistry: Chemical con artists foil drug discovery. Nature, 513, 481–483. doi: 10.1038/513481A

Dahlin, J. L., Auld, D. S., Rothenaigner, I., Haney, S., Sexton, J. Z., Nissink, J. W. M., Walsh, J., Lee, J. A., Strelow, J. M., Willard, F. S., Ferrins, L., Baell, J. B., Walters, M. A., Hua, B. K., Hadian, K., and Wagner, B. K. (2021). Nuisance compounds in cellular assays. Cell Chem. Biol., 28, 356–370. doi: 10.1016/J.CHEMBIOL.2021.01.021

Jöst, C., Nitsche, C., Scholz, T., Roux, L., and Klein, C. D. (2014). Promiscuity and selectivity in covalent enzyme inhibition: a systematic study of electrophilic fragments. J. Med. Chem., 57, 7590–7599. doi: 10.1021/JM5006918

Tikdari, A. M., and Panah, S. S. (2005). Reaction of 4-Arylidene-2-imidazolin-5-one Derivatives with 3,4-Dithio Toluene in the Presence of Triethylamine. Asian J. Chem., 17, 1527–1531.

Tomašić, T., and Mašič, L. P. (2012). Rhodanine as a scaffold in drug discovery: a critical review of its biological activities and mechanisms of target modulation. Expert Opin. Drug Discov., 7, 549–560. doi: 10.1517/17460441.2012.688743
